# Matrix vesicles from dental follicle cells improve alveolar bone regeneration via activation of the PLC/PKC/MAPK pathway

**DOI:** 10.1186/s13287-022-02721-6

**Published:** 2022-01-29

**Authors:** Genzheng Yi, Siyuan Zhang, Yue Ma, Xueting Yang, Fangjun Huo, Yan Chen, Bo Yang, Weidong Tian

**Affiliations:** 1 Engineering Research Center of Oral Translational Medicine, Ministry of Education, West China Hospital of Stomatology, Sichuan University, Chengdu, 610041 Sichuan People’s Republic of China; 2 National Engineering Laboratory for Oral Regenerative Medicine, West China Hospital of Stomatology, Sichuan University, Chengdu, 610041 Sichuan People’s Republic of China; 3 State Key Laboratory of Oral Diseases, West China Hospital of Stomatology, Sichuan University, Chengdu, 610041 Sichuan People’s Republic of China; 4 National Clinical Research Center for Oral Diseases, West China Hospital of Stomatology, Sichuan University, Chengdu, 610041 Sichuan People’s Republic of China; 5 Department of Oral and Maxillofacial Surgery, West China Hospital of Stomatology, Sichuan University, No. 14, 3rd Section, Renmin South Road, Chengdu, 610041 Sichuan People’s Republic of China

**Keywords:** Extracellular vesicles, Matrix vesicles, Osteogenesis, Dental follicle cells, Bone regeneration

## Abstract

**Background:**

The regeneration of bone loss that occurs after periodontal diseases is a significant challenge in clinical dentistry. Extracellular vesicles (EVs)-based cell-free regenerative therapies represent a promising alternative for traditional treatments. Developmental biology suggests matrix vesicles (MVs), a subtype of EVs, contain mineralizing-related biomolecules and play an important role in osteogenesis. Thus, we explore the therapeutic benefits and expect to find an optimized strategy for MV application.

**Methods:**

Healthy human dental follicle cells (DFCs) were cultured with the osteogenic medium to generate MVs. Media MVs (MMVs) were isolated from culture supernatant, and collagenase-released MVs (CRMVs) were acquired from collagenase-digested cell suspension. We compared the biological features of the two MVs and investigated their induction of cell proliferation, migration, mineralization, and the modulation of osteogenic genes expression. Furthermore, we investigated the long-term regenerative capacity of MMVs and CRMVs in an alveolar bone defect rat model.

**Results:**

We found that both DFC-derived MMVs and CRMVs effectively improved the proliferation, migration, and osteogenic differentiation of DFCs. Notably, CRMVs showed better bone regeneration capabilities. Compared to MMVs, CRMVs-induced DFCs exhibited increased synthesis of osteogenic marker proteins including ALP, OCN, OPN, and MMP-2. In the treatment of murine alveolar bone defects, CRMV-loaded collagen scaffold brought more significant therapeutic outcomes with less unhealing areas and more mature bone tissues in comparison with MMVs and acquired the effects resembling DFCs-based treatment. Furthermore, the western blotting results demonstrated the activation of the PLC/PKC/MAPK pathway in CRMVs-induced DFCs, while this cascade was inhibited by MMVs.

**Conclusions:**

In summary, our findings revealed a novel cell-free regenerative therapy for repairing alveolar bone defects by specific MV subtypes and suggest that PLC/PKC/MAPK pathways contribute to MVs-mediated alveolar bone regeneration.

**Supplementary Information:**

The online version contains supplementary material available at 10.1186/s13287-022-02721-6.

## Background

Tooth-supporting alveolar bone loss is a common consequence of periodontitis, an ongoing inflammatory disease accompanied by progressive destruction of the periodontal connective tissues [1, 2]. Up to 50% of the worldwide adult population suffers from periodontal diseases, making it one of the world’s most noteworthy diseases [3, 4]. In addition, alveolar bone loss can also be caused by post-extraction atrophy, trauma, tumor resection, and congenital abnormalities such as cleft lip and palate [5, 6]. The conventional therapies, including guided tissue regeneration (GTR), autologous bone grafting and heterogeneous bone filling, have obtained some successful cases, but commonly showed limited efficacy, prolonged therapeutic period and even secondary injuries in some complex conditions [7–9]. Additionally, mesenchymal stem cells (MSCs)-based therapies exhibited curative effects [10], while the associated inflammatory responses and safety issues seem inevitable [11–14].

It is indicated the efficacy of MSCs relies on the paracrine course and the induction of extracellular vesicles (EVs) [15, 16]. Recently, the EVs-based treatment has been proposed as a feasible cell-free therapy to manage these clinical scenarios, with the goal of regenerating lost or damaged tissues to restore normal function and structure [14, 15, 17]. EVs are a group of cell-released particles, which contain plenty of biocompatible cargos including lipids, protein, and nucleic acid, and induce the activities in intracellular communication [17]. Some studies have investigated MSC-derived EVs for alveolar bone repair [18, 19]. However, the originating cell type seems to impact the composition of EVs, and some of the EV capacities appear to vary between cell types [20–22]. Thus, utilizing cell-type-specific or tissue-specific EVs to restore damaged tissue might have intrinsic advantages and bring more therapeutic benefits.

Matrix vesicles (MVs) are a subset of EVs generated by bone-related functional cells during hard tissue development including periodontium [23, 24]. These vesicles can bind to the collagen matrix and are enveloped with mineralization-specific components such as phosphatases, calcium and inorganic phosphate [25]. The calcium (Ca) and phosphate (P) ions can either remain amorphous or can crystallize into hydroxyapatite inside MVs [26–28]. MVs are believed to mediate the accumulation of inorganic electrolytes and have a major role in the mineralization of organic matrix, which is critical for bone formation [27, 28]. In addition, MVs also have shown osteoinductive properties which indicate their therapeutic potential for bone regeneration [29, 30].

The current belief is that MSCs cultivated in media with elevated concentrations of Ca and P turn to be a reliable source of MVs [31]. MVs can be released into the extracellular space to initiate mineralization. Generally, MVs can be classified into two subtypes, media MVs (MMVs) and collagenase-released MVs (CRMVs), depending on two different isolation techniques [29, 32–35]. MMVs denote the part of MVs secreted from the cell membrane into the extracellular space acquired by direct culture media ultracentrifugation, and CRMVs represent the other part of MVs that adhere to the extracellular matrix (ECM) acquired by collagenase digestion and subsequent ultracentrifugation [32–34]. The proteomic analysis demonstrates that MMVs are enriched with plasma membrane proteins, while CRMVs mainly contain organelle and cytoskeletal proteins, indicating the different origins and vesicular attributes between them [34].

However, the previous studies mostly center on the biological properties, molecular composition and mineralization mechanism of MVs, and there is a lack of persuasive medical research and applications; thus, the therapeutic potential of MVs needs to be explicitly proved [25, 28]. In addition, most previous studies selected vascular smooth muscle cells or osteoblasts as the source for MV production [30, 35, 36], but it is known the acquisition of these cells is usually limited [37], which also impedes the translational practice of MVs. We and others have identified human dental follicle cells (DFCs), the progenitor cells contributing to the formation of the periodontium exhibited robust proliferative capacity, superior pluripotency and strong potential to construct osteogenic environments [10, 38–40]. Furthermore, DFCs are available during impact tooth extraction and without additional ethical concerns [41]. Consequently, in this study, healthy DFCs were obtained from youngsters and then be cultivated in an osteogenic differentiation condition, which may provide specific MVs adaptable to alveolar bone regeneration.

The aim of this study is to investigate the properties of DFC-derived CRMVs and MMVs in alveolar bone regeneration through in vitro and in vivo evaluations. We evaluated the ability of the different subtypes of MVs to induce cell osteogenic differentiation, modulate the expression of genes involved in bone formation processes in vitro and promote bone regeneration in vivo in rats subjected to alveolar bone defects. In particular, we assessed and compared the benefits of DFC-derived CRMVs and MMVs for new bone formation. In this study, we propose that CRMVs artificially acquired by ECM collagenase digestion and subsequent ultracentrifugation as an optimal cell-free therapy to promote alveolar bone regeneration. The final purpose of this study is the development of biocompatible and osteoinductive MVs for bone repair, especially of alveolar bone defects.

## Materials and methods

### Isolation, culture and identification of DFCs

DFCs were isolated from third molars of young patients (aging 13–20 years old) as we previously described [41, 42]. Briefly, dental follicle tissues were minced into 2–3 mm pieces and digested in collagenase type I (0.1 U/ml; Sigma-Aldrich, St. Louis, MO, USA) at 37 ℃ for 30 min. The isolated DFCs were incubated in the alpha-modified Eagle’s medium (a-MEM; Invitrogen, USA) supplemented with 10% fetal bovine serum (FBS; Gibco, USA) and 1% mixed antibiotics (100 U/mL penicillin and 100 mg/mL streptomycin, all from Sigma-Aldrich, USA). The cells were cultivated in a humidified atmosphere at 37 °C with 5% CO2, and the medium was changed every 3 days. DFCs at passages 3 to 5 were used for the following experiments.

The cell immunophenotypes were identified by the flow-cytometry essay. DFCs were incubated with antibodies against human CD24, CD29, CD31, CD44, CD90, CD106 and CD117 (all from BD Biosciences, San Jose, CA, USA) for 30 min at 4 °C in the dark and analyzed by using the Beckman Coulter Cytomics FC500 MPL system (Beckman Coulter, CA, USA). The cellular multipotent potentials in differentiation were determined according to the protocols [39]. DFCs were seeded into a 12-well plate (1 × 10^5^ per well) and cultivated to 80% confluence. Then the culture medium was changed into the osteogenic/adipogenic/neuronal induction medium and refreshed every 3 days. After 14 days of osteogenic induction, 24 days of adipogenic induction or 4 h of culture in neuronal induction, the cells were fixed in 4% paraformaldehyde and stained with Alizarin Red S, Oil Red O and nestin antibodies, respectively. Images were acquired by a confocal microscope (Olympus, USA).

### Isolation and identification of DFC-derived MMVs and CRMVs

To create an adaptable environment for MV production, DFCs were treated with the osteogenic induction medium which contains 100 mM dexamethasone, 50 μgmL^−1^ ascorbic acids (AA, Sigma) and 10 mM β-glycerophosphate (β-GP, Sigma). A moderate duration of induction (7 days) was adopted [30, 33, 36], to avoid the hypermineralization of MVs and the formation of calcified nodules which impede MV application [43, 44].

Isolation of MMVs and CRMVs was performed by two techniques [29, 31–33, 35]. MMVs were isolated from the supernatant of the osteogenic medium [30, 34, 45]. Concisely, the supernatant was spun at 2000×*g* for 30 min and 15,000×*g* for 1 h to remove cell debris and apoptotic bodies, and condensed by 100KD ultrafiltration tubes (Amicon® Ultra-15 Centrifugal Filter Units, Millipore, USA) and centrifuged at 5000×*g* for 30 min (Beckman Coulter, USA), followed by the purification of 0.8-μm filter to clear miscellaneous proteins. MMVs were finally harvested from the pellets obtained by ultracentrifugation at 150,000 × g for 1 h at 4 °C (Himac P40ST, Japan). CRMVs were acquired by collagenase digestion of DFCs and sequential centrifugation [29, 33, 35, 46]. Briefly, after 7 days of osteoinduction, DFCs were rinsed with PBS and treated with 300 U/ml collagenases (type IA, Sigma, USA) at 37 °C for 3 h. The digests were then centrifuged at 2000×*g* and 15,000×*g* to remove cell debris and apoptotic bodies. The supernatant was subsequently condensed and purified as the processes described above and eventually centrifuged at 150,000×*g* for 1 h to pellet the CRMVs (Himac P40ST, Japan). Saline was utilized to wash and resuspend the pellets of MMVs and CRMVs.

The morphology of EVs was confirmed by transmission electron microscopy (TEM). MMVs and CRMVs suspensions were applied dropwise onto formvar carbon-coated nickel grids. The grids were air-dried, stained with 2% uranyl acetate and viewed under a Hitachi H7500 TEM equipment (Hitachi H7500, Japan). Energy-dispersive X-ray (EDX) analysis (Tecnai G2 F20 S-Twin TMP, USA) was used to detect the composition and distribution of Ca/P within MVs, and zeta-potential was estimated by Phase Analysis Light Scatter (PALS, Beckman, USA), and saline was set as a blank control. The particle size and concentration of MMVs and CRMVs were determined by NTA using a ZetaView (Particle Metrix, Germany). MV markers were detected by western blotting as described below.

### Cellular uptake of MVs

According to our previously described protocol [47], green fluorescent dye DiO (Life, USA) was used to label and trace MMVs and CRMVs. Briefly, 4 × 10^4^ DFCs were co-cultured with DiO-labeled MMVs and CRMVs, respectively, for 6 h in confocal dishes. Then, DFCs were stained with phalloidin (Life, USA) and DAPI (Life Technologies, USA). Images were captured by confocal microscopy (Olympus FV1000, Japan).

### Evaluation of cell proliferation and migration

Three different concentrations of MMVs and CRMVs (5 × 10^6^ particles/ml, 5 × 10^7^ particles/ml and 5 × 10^8^ particles/ml) were used in the following in vitro experiments. The equal volume of saline was used as the control.

Cell proliferation was evaluated by a Cell Counting Kit-8 assay (CCK-8; KeyGEN, China). DFCs were seeded in 96-well plates (2 × 10^3^ cells/well) and treated with MMVs or CRMVs. 10% (v/v) CCK-8 solution was added to each well, and the cells were incubated for another 2 h at 37 °C. The optical density value was measured at 450 nm by a spectrophotometer (Thermo Fisher Scientific, USA).

Cell migration in response to MVs was assessed by the transwell migration assay and the scratch wound assay. For the 8-mm pore transwell system (Corning, USA), the upper chambers were seeded with DFCs (1 × 10^5^ cells per chamber), and the bottom wells were added with different media. After 12 h, the submembrane surface was fixed with 10% formaldehyde and stained with 1% crystal violet solution (Gene Tech, China). In scratch wound assay, DFCs were seeded in a 12-well plate (2.0 × 10^5^ /well). After confluence, the cells were scratched by a sterile pipette tip (200 μl) and then cultured with MVs for 12 h. Images of cell migration were captured using an inverted microscope (Olympus, Japan). Cell numbers or restoring percentages were counted and analyzed using the ImageJ software.

### Osteogenic induction of DFCs

DFCs were seeded in a 12-well plate (2 × 10^5^ per well) and cultured with the osteogenic medium containing different reagents. The expression of osteogenic proteins (ALP, OCN, OPN, and MMP-2) and mechanism-related proteins (PLC-γ1, PKC, ERK, p-ERK, p38, p-p38, and OCN) on day 4 was detected by western blots. Furthermore, after 14 days of induction, cells were stained with 2% alizarin red solution (Sigma-Aldrich, St Louis, MO, USA). Calcium deposition was photographed and minerals were dissolved in a 10% (w/v) cetylpyridinium chloride solution (Sangon Biotech, China) to acquire the OD value at 562 nm by the spectrophotometer.

### Analysis of protein expression

Western blot analysis was performed to determine the levels of different proteins as previously described [39]. Proteins were extracted from cell pellets or MVs using the Total Protein Extraction Kit (KeyGEN, China) and quantified using the BCA Protein Assay kits (KeyGEN, China). Aliquots of 20 μg of each sample were separated by SDS-polyacrylamide gel electrophoresis (SDS-PAGE) and transferred onto a polyvinylidene fluoride (PVDF) membrane (Millipore, USA), followed by blocking with 5% (w/v) nonfat milk for 1 h.

To identify MV markers, the PVDF membranes were incubated with primary antibodies CD63 (1:1000, Zen Bioscience, China), TSG101 (1:1000, Zen Bioscience, China), HSP70 (1:1000, Zen Bioscience, China), ALP (1:1000, Abcam, UK) and Actin (1:1000, Abcam, UK) overnight at 4 °C. To detect osteogenic markers, we used the following antibodies: ALP (1:1000, Abcam, UK), OCN (1:2000, Zen, China), OPN (1:1000, Abcam, UK), MMP-2 (1:1000, Abcam, UK) and GAPDH (1:5000, Zen Bioscience). For the investigation of signaling proteins, antibodies including PLC-γ1 (1:5000, Abcam, UK), PKC (1:2500, Abcam, UK), ERK (1:1000, CST, USA), p-ERK (1:2000, CST, USA), p38 (1:1000, CST, USA), p-p38 (1:1000, CST, USA), OCN (1:2000, Zen Bioscience, China) and GAPDH (1:5000, Zen Bioscience) were applied. Subsequently, the membranes were incubated with horseradish peroxidase (HRP)-conjugated secondary antibodies at room temperature for 1 h and chemiluminescent images were captured on an ImageQuant LAS 4000 mini system (GE Healthcare Life Sciences, USA). ImageJ software was used to determine the densitometric of the bands. The relative gray value or phosphorylation level was calculated.

### Preparation of MV-loaded collagen sponge

The CS was cut into pieces of 3 mm length (L) × 2 mm width (W) × 1 mm depth (D) and subsequently placed onto 96-well plates. 10^9^ particles of MMVs or CRMVs (about 40 μg) or 2 × 10^5^ DFCs (40 μg) suspended in 100 μl saline and dripped onto the CS overnight at 4°. The ultrastructure of samples was observed using a scanning electron microscope (SEM; Inspect F, FEI, Netherlands). The loading rate (LR_0_) was quantified by using the BCA Protein Assay kits (KeyGEN, China). According to LR_0_, the increased administration of MMVs, CRMVs or DFCs was employed to make CS virtually load the predetermined quantity.

### Preparation of alveolar bone defects and treatment of MVs

A three-dimensional (3D)-printed module was produced by a 3D printer (Zrapid, China) and composite resins to guide the creation of alveolar bone defects in vivo. Within the module, each hole was 3 mm (L) × 2 mm (W) × 1 mm (D) in size (Additional file 1: Figure S2A). The holes were separated individually for subsequent usage.

The animal experiments were reviewed and sanctioned by the Ethics Committee of Sichuan University. Eight-week-old male Sprague–Dawley rats (240–280 g) were purchased from Dashuo Experimental Animal Co. Ltd. (Chengdu, China). Referring to the previous studies [48, 49], a standard alveolar bone defect of approximately 3(L) × 2(W) × 1(D) mm was created at the right mandibular buccal alveolar bone, centering on the second mandibular molar (M2) (Additional file 1: Figure S2B). The rats were randomly assigned into 6 groups (*n* = 4 for each group). (1) Intact: healthy group without any defects; (2) Blank: control group with alveolar defect but without any treatment; (3) CS: experimental group with alveolar bone defect and treated with saline-infiltrated CS; (4) CS + MMVs: experimental group with alveolar bone defect and treated with 10^9^ MMV-loaded CS; (5) CS + CRMVs: experimental group with alveolar bone defect and treated with 10^9^ CRMV-loaded CS; (6) CS + DFCs: experimental group with alveolar bone defect and treated with 2 × 10^5^ DFC-loaded CS.

### Evaluation of the therapeutic effects

After 8 weeks, the rats were killed and the right mandibles were harvested and fixed with 10% formaldehyde. Samples were scanned by using micro-computed tomography (micro-CT; Skyscan, Bruker, Belgium) as described previously [47]. The 3D pictures were reconstructed by NRecon (Skyscan) software. The region of interest (ROI) was chosen at 3 mm (L) × 2 mm (W) × 1 mm (D) corresponding to the defect. Bone volume fraction (bone volume/total volume, BV/TV), trabecular thickness (Tb. Th), trabecular separation (Tb. Sp) and trabecular number (Tb. N) of new bone were analyzed.

After that, the mandibles were decalcified in 10% disodium ethylenediaminetetraacetic acid (EDTA, Sigma, USA), dehydrated and embedded in paraffin. The paraffin samples were prepared into 5-µm-thick sections and got Hematoxylin and Eosin (H&E), Masson’s trichrome (Baso Diagnostic Inc., China) and immunohistochemistrical staining. Primary antibodies including OPN (1:400, Abcam, UK), OCN (1:400 dilution, Zen Bioscience, China), ALP (1:200 dilution, Abcam, UK) and MMP-2 (1:250 dilution, Abcam, UK) were used. Images were acquired by an inverted microscope (Olympus, Japan). The coverage of the healing zone, the number of vessels (above 50 μm in diameter) and the average integrated density were analyzed using ImageJ software.

### Statistical analysis

Data processing and statistical analysis were conducted by SPSS 26.0 statistical software (IBM, USA). All data were presented as mean ± standard deviation. Student’s paired t test and one-way ANOVA were used to determine the level of significance. Statistical significance was set as *p* < 0.05.

## Results

### Phenotypic characteristic and multi-differentiation potential of DFCs

DFCs were characterized by a fibroblast-like morphology (Additional file 1: Figure S1A). Flow cytometric analysis showed that DFCs were positive for MSC markers (CD29, CD44, CD90) (Additional file 1: Figure S1F), but negative for hematopoietic and angiogenic markers (CD24, CD31, CD106, CD117) (Additional file 1: Figure S1G). In addition, the results of multi-differentiation induction assays indicated that DFCs were capable of osteogenic (Additional file 1: Figure S1B), adipogenic (Additional file 1: Figure S1C) and neurogenic differentiation (Additional file 1: Figure S1D). These results indicated that we had efficiently isolated and cultivated DFCs.

### Characterization of MVs

**Fig. 1:**
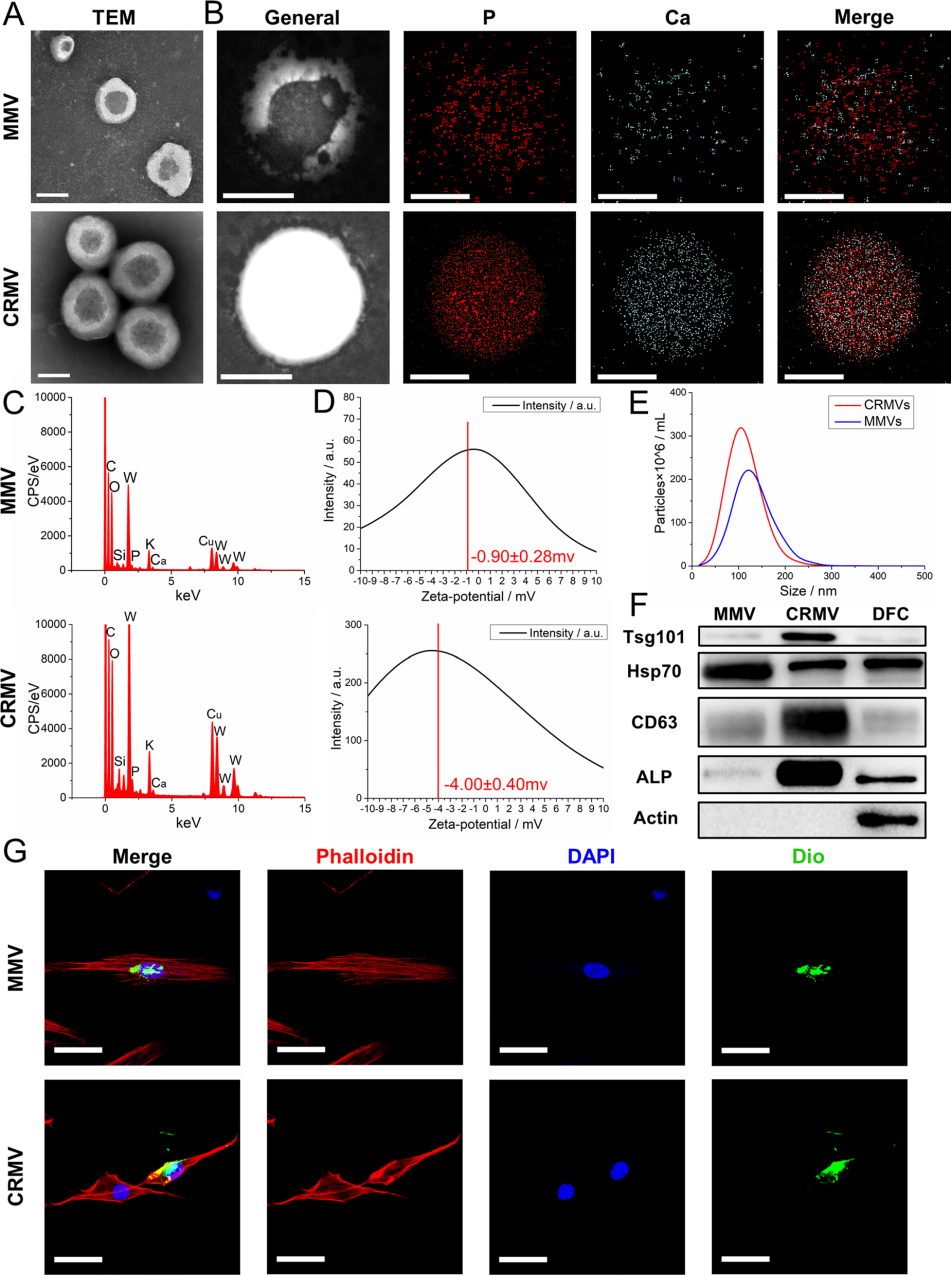
Characterization of human dental follicle cells (DFCs)-derived media matrix vesicles (MMVs) and collagenase-released matrix vesicles (CRMVs). **A** Transmission electron microscopy images of the ultrastructure. Scale bar:100 nm. Energy-dispersive X-ray analysis: **B** General views and distribution of calcium (Ca; blue) and phosphate (P; red) in MVs. Scale bar: 200 nm; **C** maps of the chemical composition of vesicles in detail. **D** Zeta-potential evaluation by the phase analysis light scatter (PALS). **E** Nanoparticle tracking analysis of particle size distribution. The red curve denotes CRMVs and blue for MMVs. **F** Western blot analysis of TSG101, HSP70, CD63, ALP and β-actin in MMVs, CRMVs and DFCs. **G** Confocal fluorescence microscopy detected the uptake of DiO-labeled MMVs or CRMVs (green) by DFCs after incubation for 6 h. Nuclei were stained with DAPI (blue). The cytoskeleton was stained with phalloidin (red). Scale bar: 20 μm

TEM revealed the EV-typical bilayer membrane ultrastructure of MMVs and CRMVs (Fig. 1A). MMVs have a typical cup-shaped appearance, while CRMVs exhibited thicker vesicle membranes and darker cores. Chemical composition inside MVs was evaluated by EDX analysis. Mineralization-associated elements including calcium (Ca), phosphorus (P) and oxygen (O) were explicitly detected (Fig. 1B). Moreover, Ca and P ions perfectly colocalized in CRMVs, while disorganized in MMVs (Fig. 1C). The zeta-potential of CRMVs (−4.00 ± 0.44mv) was quantified significantly higher than that of MMVs (-0.90 ± 0.28mv) (*p* < 0.001) (Fig. 1D). NTA showed MMVs and CRMVs shared a similar size distribution pattern and diameters approximately between 20 and 300 nm (Fig. 1E). Western blots demonstrated that both of MMVs and CRMVs expressed EV markers (TSG101, HSP70 and CD63) and were lack of cytoskeletal protein (Actin). Specifically, the transmembrane protein CD63 and the cytosolic protein TSG101 were enriched in CRMVs, while cytosolic protein HSP70 was abundant in MMVs. Of note, we found a significantly abundant expression of ALP in CRMVs, compared to MMVs and cell lysate (Fig. 1F). In summary, we successfully isolated two specific MVs from DFCs.

### Cellular internalization of MVs

To investigate whether the MVs are actively taken up by their parent cells, we performed the internalization assay. After coculturing for 6 h, MMVs and CRMVs (green) could be assembled into the cell membrane and surrounded the nuclei (Fig. 1G). These results indicated that MMVs and CRMVs could be effectively internalized by DFCs and possibly regulate cellular functions.

### MVs promoted proliferation and migration of DFCs

**Fig. 2:**
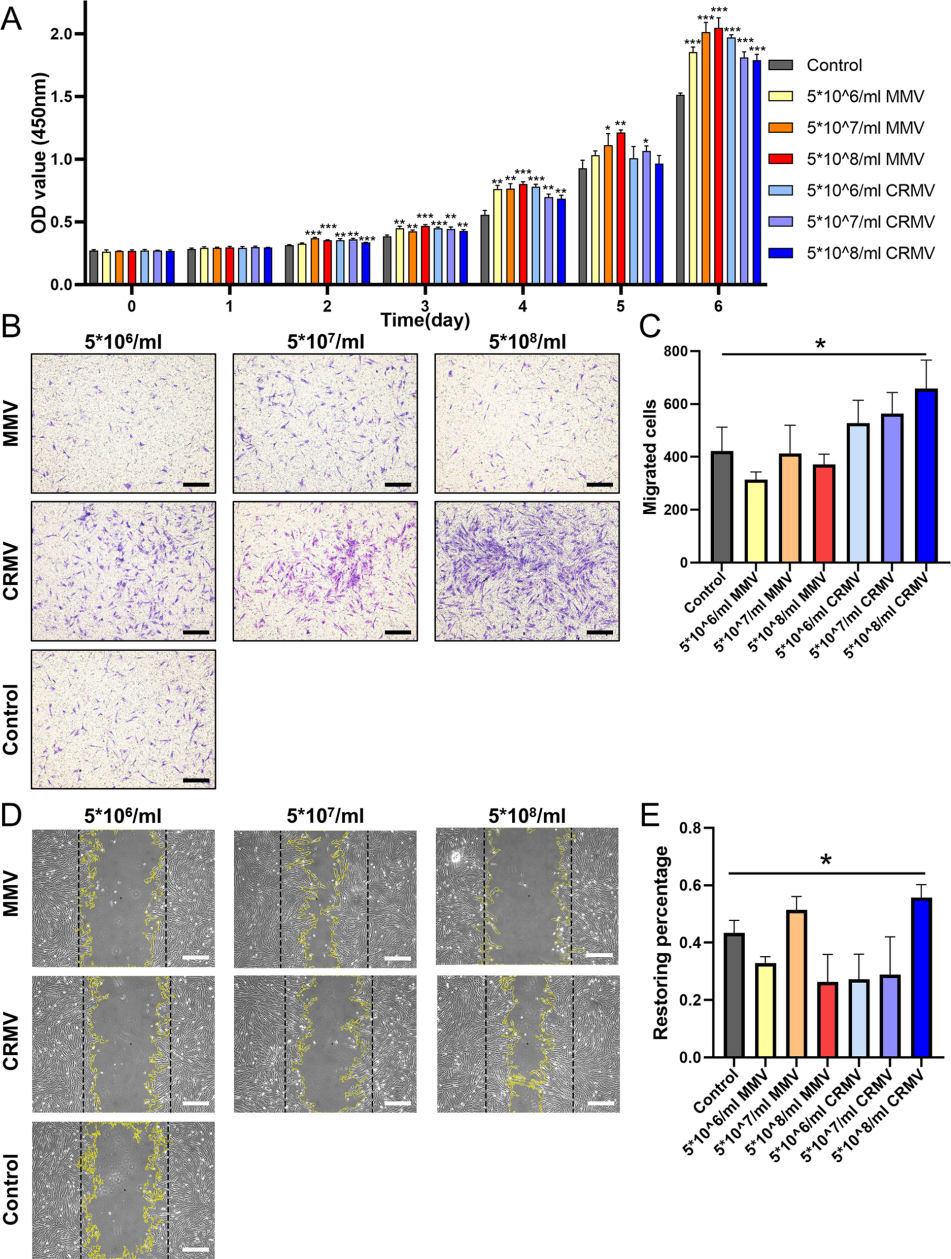
DFCs-derived MMVs and CRMVs promote the proliferation and migration of DFCs. **A** Histograms measured by the CCK-8 assay. DFCs were cultured with different concentrations (5 × 10^6^ particles/ml, 5 × 10^7^ particles/ml and 5 × 10^8^ particles/ml) of MMVs or CRMVs for 6 days. Representative microscopy images (**B**) and quantitative analysis (**C**) of cell vertical migration demonstrated by the transwell assay at 12 h. Scale bar: 200 μm. **D** Representative images of cell horizontal migration in scratch wound assay for 12 h. The black lines indicate the original state of wounds, and yellow curves underline the frontiers of the wound. Scale bar: 500 μm. **E** Restoring percentage of the recovery of wound area at the metering point. Statistical analysis is made among each experimental group and the control. *p < 0.05, **p < 0.01, ***p < 0.001

MMVs and CRMVs were arranged into three concentrations: 5 × 10^6^ particles/ml, 5 × 10^7^ particles/ml and 5 × 10^8^ particles/ml. The CCK-8 assay revealed that MVs could significantly promote DFC proliferation in a time-dependent manner (Fig. 2A). The promotion effect could be detected on day 2 and progressively amplified till day 6. Interestingly, there was no significant difference between the proliferative effect of MMVs and CRMVs, and the dose-dependent manner was not observed. As a result, we keep the predetermined concentrations in the following experiments, to know whether different tendencies would occur.

Transwell assay demonstrated CRMVs enhanced the vertical migration ability of DFCs in a dose-dependent manner (Fig. 2B). CRMVs at the concentration of 5 × 10^8^ particles/ml increased cell migration by more than a half fold compared to the control group (Fig. 2C), with a significant difference (*p* < 0.05). Scratch-wound assay indicated only the highest concentration of CRMVs (5 × 10^8^ particles/ml) resulted in increased horizontal cell migration (Fig. 2D, E), exhibiting a significant difference in comparison with the control (*p* < 0.05). However, MMVs showed no significant effects in this test. Taken together, the regulation of MVs on cell migration highly relies on the administrated dosage, and the highest concentration (5 × 10^8^ particles/ml) may bring an ideal effect for CRMVs.

### MVs enhanced osteogenic differentiation of DFCs

**Fig. 3:**
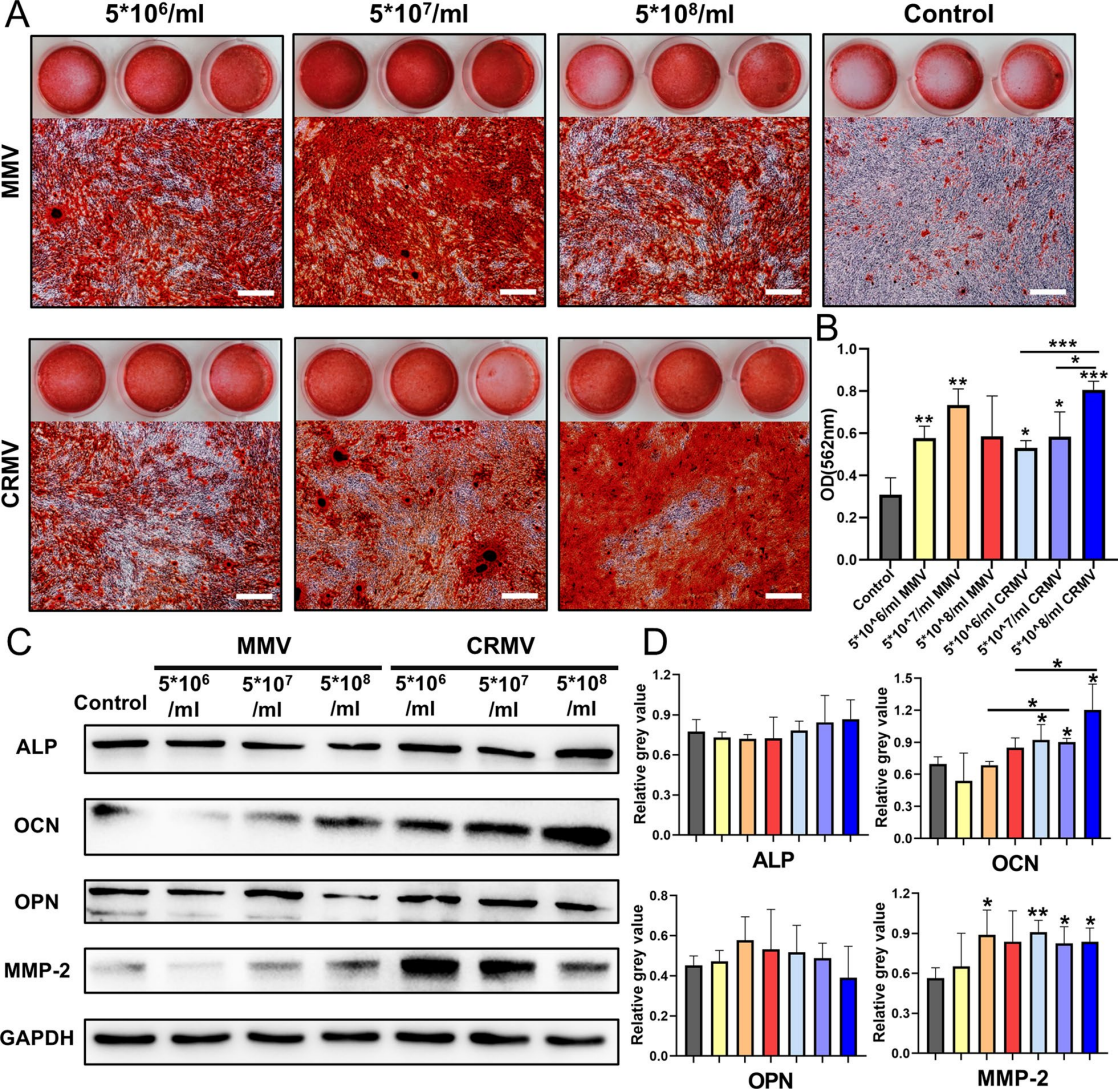
DFCs-derived MMVs and CRMVs enhance osteogenic differentiation of DFCs. **A** Representative images of alizarin red s staining of the gross view and microscopy observation after culturing for 14 days. Scale bar: 500 μm. **B** The optical density value of the dissolved mineral nodules measured by a spectrophotometer at 562 nm. Quantitative comparisons were made between each experimental group and the control. **C** Western blot analysis of osteogenic proteins (ALP, OCN, OPN, and MMP-2) in DFC lysed after the induction of different groups for 4 days, and GAPDH was set as the internal control. **D** Quantitative statistics of the related proteins. *p < 0.05, **p < 0.01, ***p < 0.001

After 14 days of osteogenic induction, numerous minerals were shown in MMVs and CRMVs induced DFCs, which were rarely found in the control (Fig. 3A, B). Moreover, the osteoinductive effects of CRMVs exhibited in a dose-dependent manner. There are statistically significant differences between the CRMV groups ((5 × 10^8^ particles/ml versus 5 × 10^6^ particles/ml (*p* < 0.001) and 5 × 10^8^ particles/ml versus 5 × 10^7^ particles/ml (*p* < 0.05)). MMVs at concentration 5 × 10^6^ particles/ml and 5 × 10^7^ particles/ml exhibited a remarkable efficacy compared to the control (*p* < 0.01).

Western blot assay was to detect the specific protein synthesis for the early-stage osteogenesis (Fig. 3C, D). The expression level of OCN and MMP-2 in DFCs was significantly increased in all CRMV groups (*p* < 0.05), though no dose-dependent manner was observed. The expression of OCN in 5 × 10^7^ particles/ml and 5 × 10^8^ particles/ml CRMV groups was significantly enhanced in comparison with the MMV groups at the same concentrations (*p* < 0.05). Variations of ALP and OPN were not intuitive in CRMVs-treated groups. Moreover, MMV groups showed no direct enhancement in the production of the osteogenic proteins. These data imply that CRMVs can markedly increase the synthesis of OCN and MMP-2 in recipient cells during the early-stage osteogenesis.

### Ultrastructure and loading efficiency of MMV, CRMV or DFC-loaded CS

**Fig. 4:**
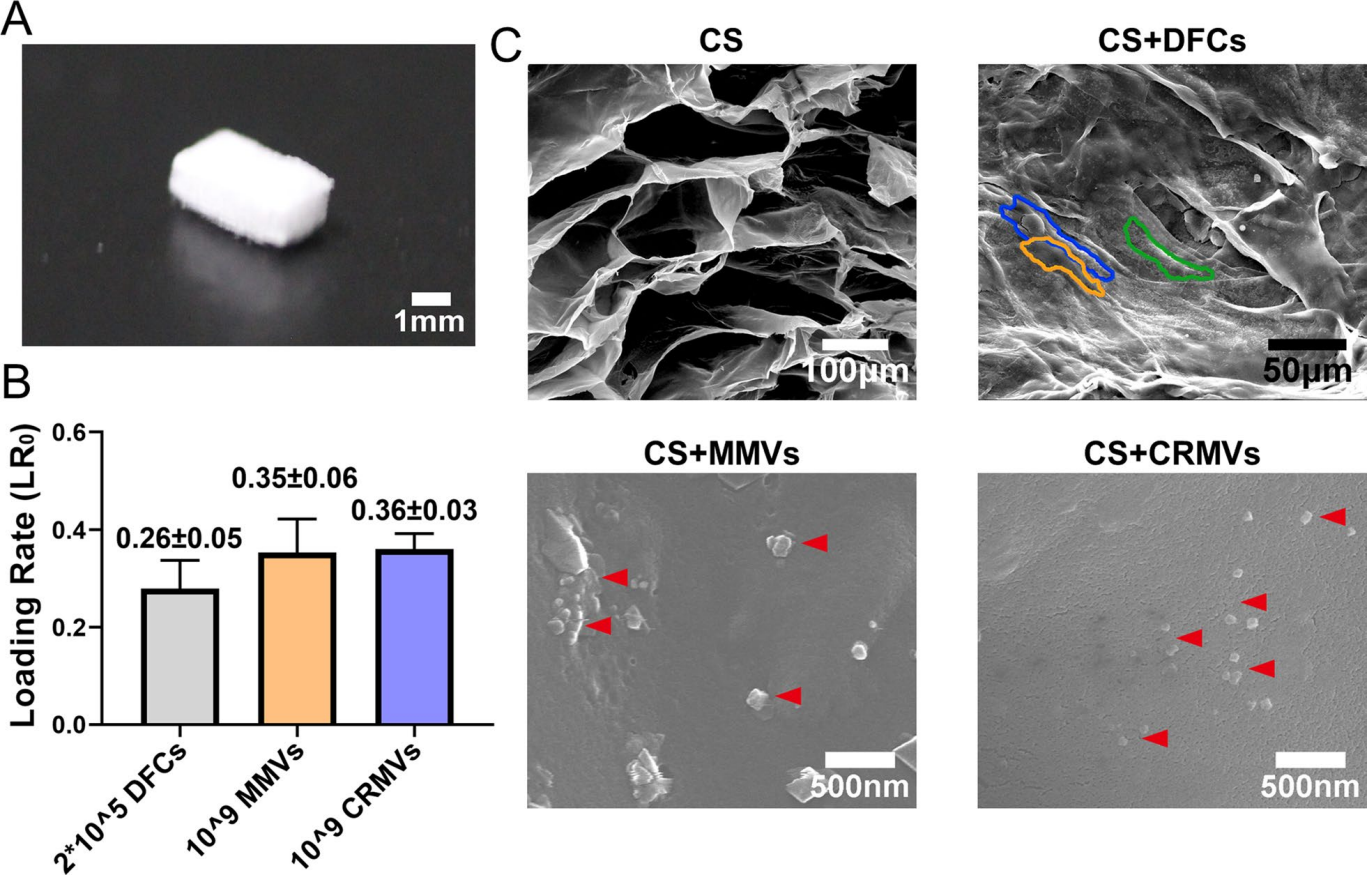
Characterization and loading efficiency of collagen sponge (CS). **A** The gross view of CS in 3 mm (L) × 2 mm (W) × 1 mm (D) size. **B** Loading efficiency of 10^9^ MMVs, 10^9^ CRMVs or 2 × 10^5^ DFCs on CS calculated by BCA Protein Assay. **C** Ultrastructure of DFC, MMV or CRMV-loaded CS and CS by scanning electron microscope. DFCs were marked by colored lines (blue, yellow or green). Scale bars are shown in the graphs, individually

CS was cut into pieces of 3 mm (L) × 2 mm (W) × 1 mm (D) (Fig. 4A). Since the biological effects of CRMVs were confirmed to correlate with a dose-dependent manner in the above results, we applied the highest feasible dose. 1 × 10^9^ MMVs, 1 × 10^9^ CRMVs or 2 × 10^5^ DFCs (with the equivalent content of protein of MVs) were loaded on the CS, and the loading efficiency was calculated. The results showed LR_0_ was (0.26 ± 0.05) for DFCs, (0.35 ± 0.06) for MMVs and (0.36 ± 0.03) for CRMVs (Fig. 4B). According to the LR_0_, the dosage of droplets in each group was adjusted to make the CS load the predetermined quantity.

Subsequently, the ultrastructure of different samples was imaged by SEM (Fig. 4C). The CS exhibited a typical sponge-like structure, with an organized frame and regular distribution of pores. In DFC-loaded CS, cell nucleus and cytoplasmic protrusions were observed and profiled by colored lines (blue, yellow and green). In MMV and CRMV-loaded CS, vesicles with a diameter around 100 nm adhered to the material were observed. These findings demonstrated the feasibility of CS as a carrier of DFCs, MMVs and CRMVs.

### CRMVs promoted alveolar bone defect regeneration

**Fig. 5:**
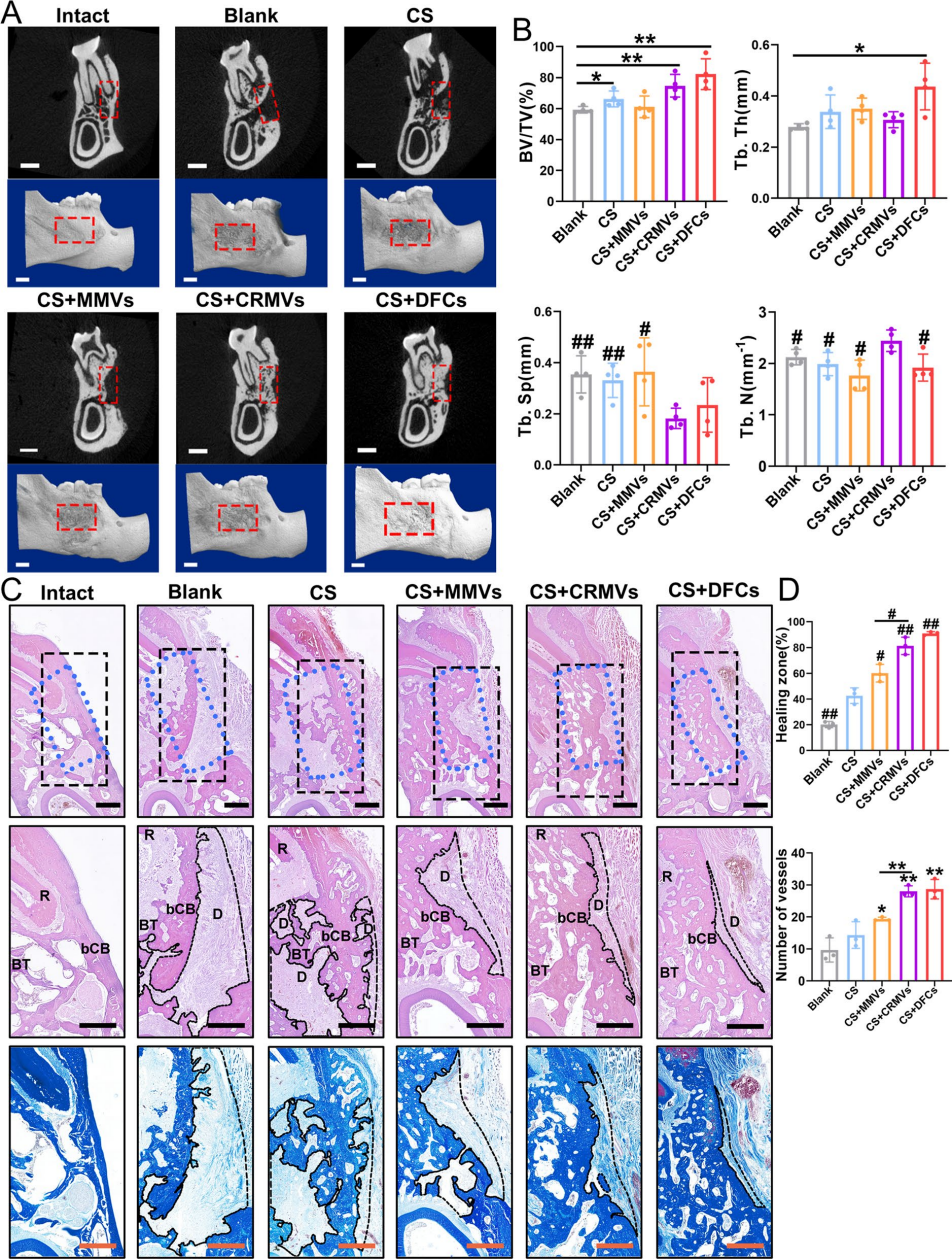
CRMV-loaded CS shows remarkable therapeutic effects in alveolar bone regeneration of SD rats after 8 weeks of administration. **A** Three-dimensional reconstruction images and two-dimensional section graphs of the microcomputed tomography, focusing on the second mandibular molar. Scale bar: 1 mm. **B** Diagrams of the indexes including bone volume/total volume, trabecular thickness, trabecular separation and trabecular number. *n* = 4 per group. *p < 0.05, **p < 0.01. ^#^*p* < 0.05, ^##^*p* < 0.01 (versus the CS + CRMVS). **C** Representative images of the healing status of alveolar bone in histological observations. (Line 1) Hematoxylin and Eosin staining at 40 × magnification about second mandibular molar at coronal section. The blue dotted boxes represent the original area of defects, and black boxes express the region of interest (ROI). Scale bar: 500 μm. (Line 2) Images of ROI at 60 × magnification. **D**, Unhealing defects. bCB, buccal cortical bone. BT, bone trabecula. R, tooth root. Scale bar: 500 μm. (Line 3) Representative images of Masson staining within ROI at 60 × magnification. Scale bar: 500 μm. **D** The sizes of newborn bone were measured, including the healing rate of the center and the number of vessels (over 50 μm in diameter). *p < 0.05, **p < 0.01. ^#^*p* < 0.05, ^##^*p* < 0.01 (versus the CS)

Eight weeks after implantation, the right mandibles of different groups (Blank, CS, CS + DFCs, CS + MMVs and CS + CRMVs) were harvested. Samples were firstly analyzed by micro-CT scanning. The 3D reconstructed images showed the general view of the tissue blocks and the defect area on the sagittal plane, and the 2D topographical section images illustrated the state of defects from the coronal plane of the M2 (Fig. 5A). The ROI (indicated by red dotted boxes) was determined centering on the M2, with the equal area of the initial defect (3 × 2 × 1 mm). Within the groups (Blank, CS and CS + MMVs), defects were poorly repaired, and there were still significant cracks. In contrast, for CS + CRMVs and CS + DFCs, the damaged tissues have been recovered into a good state, without large pieces of hole zone remaining.

The quantitative analysis provided more details (Fig. 5B). In the Blank, CS and CS + MMVs, BV/TV was calculated at around 60%. CS + CRMVs had notably improved the level of BV/TV (over 75%), which was superior to the above groups (*p* < 0.01 versus the Blank), evaluated approximately to the CS + DFCs (around 80%). In particular, the CS + CRMVs were associated with the largest value of Tb. N (about 2.5/mm) and the lowest level of Tb. Sp (less than 0.2 mm) among all the groups, and the two statistical metrics showed a significant difference in comparison with other groups (Blank, CS and CS + MMVs; *p* < 0.05). However, the above two indicators of DFCs-treated groups were not remarkable, which only presented a relatively high level of Tb. Th (*p* < 0.05 versus the control). These proofs revealed CRMVs could effectively stimulate the formation of new bones to an ideal extent and reconstruct bone trabeculae into a good status, which brought more benefits than MMV-loaded CS or CS alone.

Tissue sections were prepared in coronal orientation, centering on the M2. The blue dotted boxes represented the original area of defects, and black boxes expressed the ROI (Fig. 5C). Within ROI, the regenerated alveolar bone involved two portions: buccal cortical bone (bCB) in the lateral and bone trabeculae (BT) in the central, and other regions without recovery were regarded as unrepaired defects (D). In HE staining and Masson staining, CS + CRMVs and CS + DFCs showed the most notable effects for regeneration, in which the new bone has filled most of the defect area and various bone trabeculae formed. The bCB attained a favorable recovery, presenting as complete and continuous, and BT was well remodeled into symmetrical and compact structures. In contrast, Blank and CS exhibited a wide area of unrepaired defects and an irregular morphology as well as limited numbers of BT. Furthermore, CS + MMVs showed a moderate curative effect, which presented a regular state of BT but limited healing of bCB. Quantitative analysis also showed within the healing frontier, the restoring rate and number of vessels in CS + CRMVs and CS + DFCs were superior to the other groups (Blank, CS and CS + MMVs; *p* < 0.05) (Fig. 5D).

**Fig. 6:**
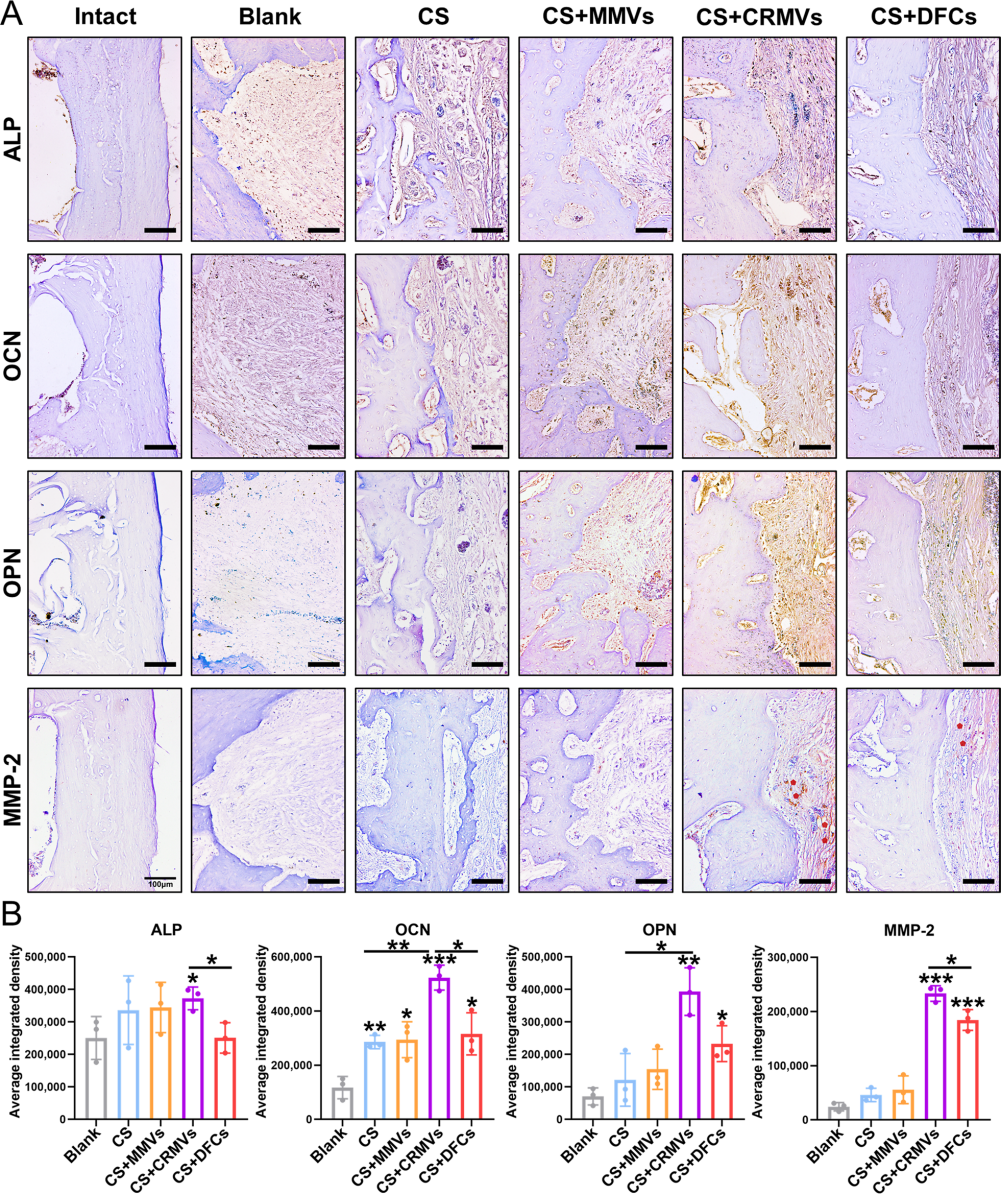
Immunohistochemistrical evaluation of the frontier of the defects. **A** The expression of osteogenic proteins (ALP, OCN, OPN and MMP-2) was detected and the brown indicated positive sites. The red pentagons indicated positive regions of MMP-2. Scale bar: 100 mm. **B** Quantitative analysis of the expression level of the above proteins. *p < 0.05, **p < 0.01, ***p < 0.001

To further investigate the osteogenic activity of the frontier of the defects, the expression of osteogenic proteins (ALP, OCN, OPN and MMP-2) was assessed by immunohistochemistrical staining (Fig. 6A, B). For the Blank, the above proteins were weakly expressed and limited in concentration. CS and CS + MMVs were able to induce an increase in OCN production (*p* < 0.05), and CS + DFCs could further intensify the expression of OPN and MMP-2 (*p* < 0.05). It was remarkable that CS + CRMVs had an outstanding capacity of promoting OCN and MMP-2 synthesis in recipient tissues (*p* < 0.001), which was measured in more quantify than DFCs-treated ones (*p* < 0.05). Moreover, CRMVs also brought a significant augment in ALP and OPN expression (*p* < 0.05 versus the Blank).

The results of in vivo experiments confirmed that CRMVs possessed favorable osteoinductive potency, and could promote osteogenesis of alveolar bone defects, bringing potent results for cell-free treatment. Additionally, the biological activity of CRMVs in osteogenic protein regulation has been verified both in vitro and in vivo.

### CRMVs activated osteogenesis via the PLC/PKC/MAPK pathway

The underlying molecular mechanism was subsequently explored to account for the distinct regenerative outcomes among the two MVs. Since MVs can be internalized by DFCs and promote the upregulation of osteogenic proteins, a possible PLC/PKC/MAPK (Phospholipase C, PLC; Protein kinase C, PKC; Mitogen-activated protein kinase, MAPK) pathway associated with exogenous substance uptake and osteogenic stimulation is assumed to be involved. We found that CRMVs at 5 × 10^8^ particles/ml exhibited superiority in cell migration and osteogenic differentiation, while MMVs at the same concentration exhibited limited effects. To explore, in depth, the reasons for the difference, we selected the concentration of 5 × 10^8^ particles/ml in the mechanism experiments.

**Fig. 7:**
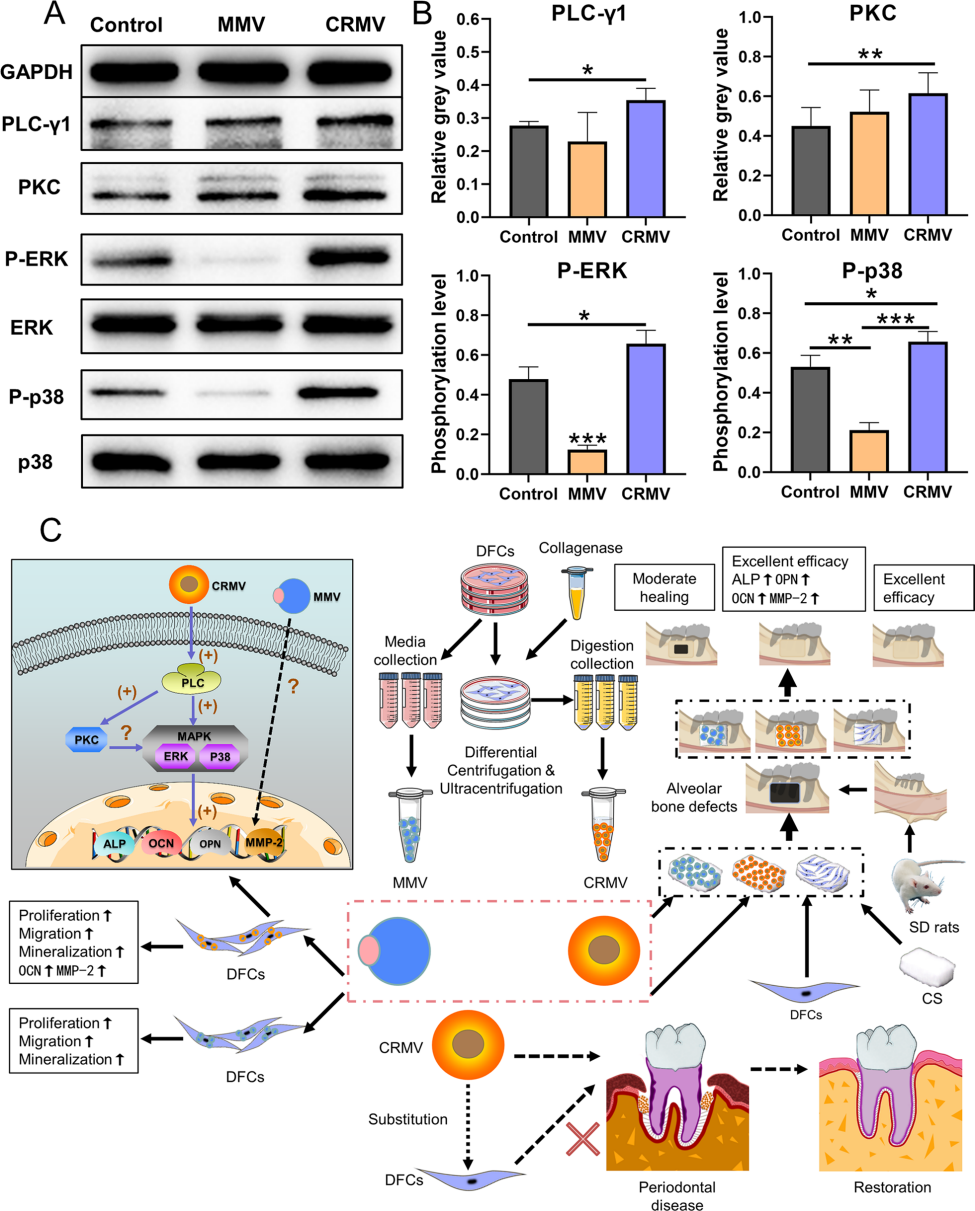
DFCs-derived CRMVs enhance osteogenic differentiation of DFCs via activating the PLC-PKC-MAPK pathway. **A**, **B** Western blot analysis demonstrated the expression of the proteins involved in the PLC-PKC-MAPK pathway in DFCs, after the incubation of 5 × 10^8^ particles/ml MMV or CRMV for 4 days. **A** Representative images of the involved proteins (PLC-γ1, PKC, ERK, p-ERK, p38 and p-p38). **B** Quantitative evaluation on the expression state or phosphorylation level of the above proteins, GAPDH was served as the internal control. *p < 0.05, **p < 0.01, ***p < 0.001. **C** Schematic illustration of the isolation, functional examinations, periodontal therapeutic benefits and osteoinductive mechanism of DFCs-derived MMVs and CRMVs

As expected, after culturing with 5 × 10^8^ particles/ml CRMVs for 4 days, the expression of PLC-γ1 in DFCs was elevated (*p* < 0.05), along with the increased expression of PKC (*p* < 0.01) and upregulated phosphorylation level of ERK and p38 (*p* < 0.05) (Fig. 7A, B). DFCs treated with 5 × 10^8^ particles/ml MMVs also brought a moderate enhancement of expression of PKC, but suppressed phosphorylation levels of ERK and p38 (*p* < 0.05) without significant influence on PLC-γ1. Taken together, these results demonstrated that CRMVs stimulated the PLC/PKC/MAPK pathway to regulate osteogenesis (Fig. 7C), and MMVs failed to function in this field.

## Discussion

As a part of tooth-supporting tissue, alveolar bone secures the stability of the periodontal tissues, provides the blood supply of the tooth and constructs a community of periodontium with connecting periodontal components [1, 2, 50]. In this regard, the well-repair of alveolar bone loss caused by periodontal diseases or other pathological stimuli is of great significance to ensure the structural integrity and functional coordination of periodontal tissues [51]. To date, there is still an unmet need for novel cell-free regenerative therapies, which are expected to bring favorable prognoses and avoid the problems associated with the direct administration of stem cells [14, 17]. This study demonstrated that DFC-derived CRMVs significantly strengthened alveolar bone regeneration and achieved a comparative therapeutic level of MSCs-based treatment in a murine periodontal defect model. Specifically, CRMVs possessed biological attributes adaptable for mineralization and strong potency in osteoinduction. They promote the ossification of target cells and tissues through activating the PLC/PKC/MAPK pathway. These findings provide a new preferred cell-free strategy for periodontal regenerative treatments.

MVs present as tissue-specific EVs for biomineralization and osteogenesis, which are theoretically classified into MMVs and CRMVs. Some studies revealed the biological effects of MVs on bone-related functional cells associated with increased osteogenic gene expression, indicating their potential to promote cellular mineralization [29, 30, 43]. To explain more intuitively, we examined these characteristics of MVs in vitro. We discovered both MMVs and CRMVs could elicit extensive calcium deposition of DFCs by approximately two- to threefold in quantity compared to the control. Moreover, this trend was still found even employing MVs at very low concentrations. The results suggest a strong potency of MVs to facilitate osteogenesis, which probably endows them with irreplaceable use value for alveolar bone regeneration.

The favorable findings in vitro drove us to explore the medical role of MVs in alveolar bone restoration in vivo. To repair the defect at 3 × 2 × 1 mm, MMVs, CRMVs or DFCs were routinely loaded by CS and implanted in situ for a period of eight weeks. Afterward, a notable bone healing zone with abundant blood supply was induced by CRMVs, which filled most areas of the traumatic cavity and was as efficient as the DFCs-treated individuals. Exclusively, CRMVs could result in a visible formation of dense and well-defined bone trabeculae of the new bone and was in conjunction with the upregulation of a series of osteogenesis-related proteins within the healing frontier, superior to the other groups in related measures. Interestingly, the MMVs-treated groups presented a disparity in the status of bone healing rate, which did not exert significant improvement in the above assessments. These observations suggest CRMVs can bring significant rescuing effects and should be an optimized promoter for alveolar bone regeneration. Although recent studies have reported the regenerative potential of chemical synthesized MVs in calvaria defects [52], for the moment, the in vivo therapeutic effects of organism-derived MVs remain largely unknown [25]. To our knowledge, this is the first study to evaluate the bone-regenerative capacity of biogenic MVs among different origins.

Since MVs are particles generated by bone functional-related cells, they have the opportunity to inherit specific cellular substances and play a role in the execution of biomineralization and osteogenesis [27]. The current findings indicate the bone regeneration capacity of MVs is potentially attributed to three major fields: (1) ability to recruit electrolytes and facilitate mineral formation; (2) upregulation effect in protein expression targeting ossification; (3) unique performance to elicit bone trabeculae neogenesis [25, 28, 53].

Biomineralization is confirmed to be a fundamental step for bone formation, which mainly manifests as the recruitment of calcium and phosphate and crystal deposition, and this complex activity highly relies on specific proteins [54]. MVs are identified to be native carriers of ion channel proteins and enzymes, which harness the evolution of biomineralization through the regulation in electrolyte transport and chemical reactions. In this study, we identified the component of alkaline phosphatase (ALP) in the two MVs. Other proteins include sodium/phosphate cotransporter type III (Pit1; phosphate channel protein), calcium channel proteins (Annexin; Anx), ecto-nucleotide pyrophosphatase/phosphodiesterase 1 (ENPP1) were also reported in prior studies [26, 54, 55]. Nevertheless, among the cargoes MVs carry, ALP is considered a pivotal enzyme with multi-dimensional importance. Previously, Bolean et al. examined the property of synthesized liposomes enriched with ALP or Anx family proteins under osteogenic conditions and found measurable biomineralization in ALP-abundant liposomes but barely detectable in AnxA5-abundant ones, which suggested the leading role of ALP [56]. ALP is a peripheral membrane protein attached to the outer leaflet of MVs [57]. Relying on a glycosylphosphatidylinositol (GPI) anchor, ALP can build a chemical bonding between MVs and collagen fibrils in ECM, which secures the stability necessary for inducing mineralization [58]. In this condition, ALP abundant in MVs critically enhances mineral deposition through transforming inorganic pyrophosphate (PPi) into inorganic phosphate, which is an essential substrate for the whole mineralization process [54]. Furthermore, it is proposed that ALP participates in the extracellular ADP and ATP synthesis and provides vital energy for the following mineralization activities [59]. In this study, significant enrichment of ALP was determined within CRMVs instead of MMVs, indicating CRMVs may possess stronger potentials to accelerate mineral deposition. The following investigations of elementary distribution among the two particles verified this speculation. Our results showed the distribution of Ca and P overlapped in CRMVs on a large scale, but randomly arrayed in MMVs. Evidence proved that the distribution of the two elements correlates to the evolutional phase of crystals. A universal colocation of Ca and P indicates the minerals reach the mature stage, while an irregular array means the early stage [26, 35]. Therefore, it implies that compared to MMVs, CRMVs are more apt to accelerate mineral accumulation relying on the higher concentration of ALP.

Exogenous therapeutic agents which potentiate cellular phenotypic changes and protein secretion specific for osteogenesis can greatly support bone regeneration [60]. The upregulated functional proteins can subsequently direct the degradation of organics in the matrix and induce crystal deposition, ultimately promoting new bone formation [61]. It is previously reported the bone regenerative effect of chemical synthesized MVs correlated to their upregulating actions on Runx2, a key factor for the early mineralization [52]. Consequently, we investigated the biogenic MV-mediated modification of gene expression in the recipient cells and targeted tissues. In this context, CRMVs greatly elevated the expression level of osteogenic marker proteins including matrix metalloproteinases-2 (MMP-2) and osteocalcin (OCN) in DFCs only after the co-culturing for 4 days with any predetermined concentrations. This positive effect could also be found in the healing frontier of CRMVs-treated defects, which exhibited a significant augment in all interested proteins. MMP-2 is regarded as secreted regulatory agents in the matrix, which remodels the ECM components, enhances protein absorption and provides the space to accommodate minerals, thus advancing the process of ossification [39, 62]. MMP-2 also has been proposed to assist the release and activation of neighboring growth factors and contribute to osteoblastic differentiation [27]. OCN is considered as a classical osteogenic marker reflecting the mature osteogenic phenotype [63]. Furthermore, OCN influences the hydroxyapatite nucleation in virtue of the high affinity for calcium ions, which induce mineralization and subsequently compose the matrix of new bone [64, 65]. A marked increase in OCN synthesis portends the complete success of ossification. However, MMVs underperformed in osteogenic protein regulation, which revealed a limited biological activity. Physical properties among these vesicles potentially provide some clues for this distinction. Zeta-potential is an indicator of the surface potential of biomolecules, which reflects the magnitude of repulsion force between individual particles [66, 67]. In general, the small zeta-potential is usually associated with insufficient intermolecular repulsion force and incurs molecular aggregation [68]. Since EV samples with smaller zeta-potential are prone to aggregation, particles with higher charges are less likely to aggregate and much more stable [69] and can probably gain more opportunities to interplay with target cells [68]. Considering that, we evaluated and observed a higher value of zeta-potential affiliated to CRMVs, which may render them more predominant mediating effect and osteogenic-inductive ability in medical treatment. Moreover, some unknown mechanisms probably influence the inductive activity of MVs during this course, which warrants further exploration.

Bone trabeculae manifest as indispensable elements of the vertebrate skeleton system and alveolar bone within a body, which shape like a small beam, strut or rod and possess vital importance for the entire bone structure [70]. In the composition of bone trabeculae, some transcription factors, including insulin-like growth factor (IGF), transforming growth factor-β (TGF-β), fibroblast growth factor (FGF) and epidermal growth factor (EGF), have been examined [71]. These regulators are likely to be delivered into the unhealing zones and stimulate the proliferation of regional cells, thus enhancing the repair procedure. Moreover, bone trabeculae also serve as the reservoir of readily available calcium during the daily circulatory activity [72], which can create an adaptable environment for the bone cortex restoration through the supply of calcium [73]. In this study, CRMVs were found to promote the neogenesis of bone trabeculae, in correlation with a notable remodeling of the buccal cortical bone and broad healing coverage of the defects, which verifies a good restoration of bone trabeculae can indeed benefit the integral regeneration process of the alveolar bone. It is evidenced that MVs can participate in the trabecular bone development by recruiting minerals and directly integrating themselves with the bone matrix [53, 74]. The previous proteomic analysis demonstrates that CRMVs are enriched with a group of factors that probably contribute to their integration, which are comprised of fibrillin, type VI collagen, proteoglycan link protein and so forth [46]. In contrast, MMVs are comprised of plasma membrane proteins without significant functions in the evolution of bone trabeculae [34]. Collectively, in virtue of the various connective proteins, CRMVs are more prone to stimulate trabecular bone reconstruction and support the framework of alveolar bone regeneration.

To further understand the functional roles of MVs in osteogenesis, we tried to explore the possible molecular mechanism. PLC is a family of membrane-associated enzymes, which is known for its great significance in the conduction between cell internalization and signal transduction [75, 76]. PLC receives signals originating from cell endocytosis [75–77]. Once triggered, PLC could result in the remodeling of the plasma membrane and elicit the production of multiple secondary messengers such as PKC and MAPK cascade (involving ERK and p38), finally regulating osteogenic protein expression and cell metabolism [76–79]. Some proteomic studies have determined the presence of PLC in CRMV contents [26, 80]. However, there are infrequent reports that focused on the biological state of PLC and PKC in MVs-treated cells [77]. Since the cell endocytosis of MVs was detected in the present study, PLC contained in CRMVs was likely to be assimilated by the target cells and stimulate the following activities. Therefore, we postulated CRMVs yielded osteoinductive effects, thereby activating the PLC/PKC/MAPK pathway, and PLC-γ1 was selected as a representative subpopulation to illustrate. Consistent with our hypothesis, the results showed CRMVs were able to induce the sequential activation of the PLC/PKC/MAPK pathway. In contrast, although MMVs exhibited a moderate activation of PKC, the enhancement of PLC and MAPK could not be detected, which may account for their insufficiency in regenerative potential. Linking these results with the performances in protein modulation by the two MVs, we speculate that the activation of PLC and subsequently, MAPK is necessary for the upregulation of osteogenic proteins, and this effect may bypass the PKC-dependent route. Moreover, some unknown mechanisms related to the independent regulation of MAPK by PLC need to elucidate in further investigations.

Recently, the immunomodulatory property of EVs during tissue repair has been explored. EVs can transfer their contents, including protein and lipids to immune cells [81], which alleviate inflammation and assist regenerative activities during bone healing [82, 83]. MVs are also involved in some studies regarding immune regulation. The Bessueille’s group found in the growth plate cartilage of ALP-deficient transgenic mice, the expression level of IL-1β, IL-6 and some pro-inflammatory agents was significantly increased. In response, chondrocytes accelerated the synthesis of MVs with many biomolecules, in which ALP was able to stimulate IL-10 secretion in neutrophils to balance the homeostasis and alleviate the inflammatory damages [58]. MVs are also effective inhibitors in osteoclast formation. It is demonstrated that during the co-culture process, MVs derived from osteoblasts could decrease the transcription of Nfatc1, Dcstamp and Ctsk in monocytes [43]. Since the above genes are accepted as adjuvants that guide osteoclast and resorption pit formation, MVs turn to be a defender of hard tissues free from bone resorption. Additionally, under the systemic inflammatory condition, it is reported that the liver can strategically produce a group of immunoregulatory factors, fetuin-A for instance. Through the endovascular approach, Fetuin-A clusters were then delivered to the peripheral tissues suffering from inflammation. Some of these proteins would timely induce the release of cationic polyamines and high mobility group box-1 (HMGB1), exerting critical regulations on inflammation [84], and the other portions would be stored in MVs producing cells or in MVs, which potentially act as a reservoir [34, 85].

It is undeniable although the alveolar bone defects have been well-repaired by MVs-based strategies, several improvements are expected for future studies. The therapeutic potential of MVs requires more persuasive investigations in large animals (e.g. beagle dogs and rhesus monkeys), whose physiological structures are more analogical to humans. In future studies, we are also enthusiastic to investigate the immunomodulatory effects of MVs, and expect they have a dual role in osteoinduction and immunoregulation. The functional mechanism and administration approach associated with MVs still need further exploration to maximize their medical practice as a reliable therapy for clinical periodontal treatment.

## Conclusion

In our current study, MMVs from DFCs culturing media and CRMVs from the collagenase-digested DFCs suspension were smoothly isolated and could be internalized inside cells. DFC-derived MMVs moderately and CRMVs notably enhanced cell viabilities including proliferation, migration and osteogenic differentiation of DFCs. Furthermore, in alveolar bone regeneration, CRMVs possess more osteoinductive advantages in comparison with MMVs and can be regarded as a promising candidate for stem cell-based treatment for periodontal disease. The underlying reason is probably CRMVs can activate the PLC/PKC/MAPK pathway, which contributes to the ultimate upregulation of osteogenesis-related gene expression (Fig. 7C). The effect of MMVs warrants further investigations. Thus, the results in the present study also provide orientations to investigate the medical benefits of MVs in-depth in the future.

## Supplementary Information

**Additional file 1**:** Figure S1**. Characterization of dental follicle cells (DFCs). **Figure S2**. The creation of the alveolar bone defects in the SD rat guided by a module.

## Data Availability

The data that support the findings of this study are available from the corresponding author upon reasonable request.

## Abbreviations

EVs: Extracellular vesicles
MVs: Matrix vesicles
DFCs: Dental follicle cells
MMVs: Media MVs
CRMVs: Collagenase-released MVs
CS: Collagen sponge
GTR: Guided tissue regeneration
MSCs: Mesenchymal stem cells
Ca: Calcium
P: Phosphate
O: Oxygen
ECM: Extracellular matrix
FBS: Fetal bovine serum
a-MEM: Alpha-modified Eagle’s medium
AA: Ascorbic acids
β-GP: β-Glycerophosphate
TEM: Transmission electron microscopy
EDX: Energy-dispersive X-ray
NTA: Nanoparticle tracking analysis
PALS: Phase Analysis Light Scatter
CCK-8: Cell Counting Kit-8
SDS-PAGE: SDS-polyacrylamide gel electrophoresis
PVDF: Polyvinylidene fluoride
ALP: Alkaline phosphatase
OCN: Osteocalcin
OPN: Osteopontin
MMP-2: Matrix metalloproteinase-2
GAPDH: Glyceraldehyde-phosphate dehydrogenase
HRP: Horseradish peroxidase
SEM: Scanning electron microscope
LR_0_: Loading rate
3D: Three-dimensional
M2: Second mandibular molar
Micro-CT: Micro-computed tomography
ROI: Region of interest
BV/TV: Bone volume/total volume
Tb. Th: Trabecular thickness
Tb. Sp: Trabecular separation
Tb. N: Trabecular number
EDTA: Ethylenediaminetetraacetic acid
H&E: Hematoxylin and Eosin
bCB: Buccal cortical bone
BT: Bone trabecular
PLC: Phospholipase C
PKC: Protein kinase C
MAPK: Mitogen-activated protein kinase
Pit1: Sodium/phosphate cotransporter type III
Anx: Annexin
ENPP1: Ecto-nucleotide pyrophosphatase/phosphodiesterase 1
GPI: Glycosylphosphatidylinositol
PPi: Inorganic pyrophosphate
IGF: Insulin-like growth factor
TGF-β: Transforming growth factor-β
FGF: Fibroblast growth factor
EGF: Epidermal growth factor
HMGB1: High mobility group box-1

## References

[1] Xiong J, Gronthos S, Bartold PM (2013). Role of the epithelial cell rests of Malassez in the development, maintenance and regeneration of periodontal ligament tissues. Periodontol.

[2] Yu T and OD Klein. (2020). Molecular and cellular mechanisms of tooth development, homeostasis and repair. Development 147:dev184754. 10.1242/dev.184754 PMC6983727 31980484

[3] Darveau RP (2010). Periodontitis: a polymicrobial disruption of host homeostasis. Nat Rev Microbiol.

[4] Hajishengallis G (2015). Periodontitis: from microbial immune subversion to systemic inflammation. Nat Rev Immunol.

[5] Izumi Y, Aoki A, Yamada Y, Kobayashi H, Iwata T, Akizuki T, Suda T, Nakamura S, Wara-Aswapati N, Ueda M, Ishikawa I (2011). Current and future periodontal tissue engineering. Periodontol.

[6] Kononen E, M Gursoy and UK Gursoy. (2019). Periodontitis: a multifaceted disease of tooth-supporting tissues. J Clin Med 8. 10.3390/jcm8081135 PMC6723779 31370168

[7] Chen FM, Jin Y (2010). Periodontal tissue engineering and regeneration: current approaches and expanding opportunities. Tissue Eng Part B Rev.

[8] Dissaux C, Wagner D, George D, Spingarn C, Remond Y (2019). Mechanical impairment on alveolar bone graft: a literature review. J Craniomaxillofac Surg.

[9] Chrcanovic B, Gomez R (2019). Gingival cyst of the adult, lateral periodontal cyst, and botryoid odontogenic cyst: an updated systematic review. Oral Dis.

[10] Guo S, Kang J, Ji B, Guo W, Ding Y, Wu Y, Tian W (2017). Periodontal-derived mesenchymal cell sheets promote periodontal regeneration in inflammatory microenvironment. Tissue Eng Part A.

[11] Chen FM, Gao LN, Tian BM, Zhang XY, Zhang YJ, Dong GY, Lu H, Chu Q, Xu J (2016). Treatment of periodontal intrabony defects using autologous periodontal ligament stem cells: a randomized clinical trial. Stem Cell Res Ther.

[12] Ferrarotti F, Romano F, Gamba MN, Quirico A, Giraudi M, Audagna M, Aimetti M (2018). Human intrabony defect regeneration with micrografts containing dental pulp stem cells: a randomized controlled clinical trial. J Clin Periodontol.

[13] Ren K (2019). Exosomes in perspective: a potential surrogate for stem cell therapy. Odontology.

[14] Novello S, Pellen-Mussi P, Jeanne S (2021). Mesenchymal stem cell-derived small extracellular vesicles as cell-free therapy: Perspectives in periodontal regeneration. J Periodontal Res.

[15] Pashoutan Sarvar D, Shamsasenjan K, Akbarzadehlaleh P (2016). Mesenchymal stem cell-derived exosomes: new opportunity in cell-free therapy. Adv Pharm Bull.

[16] Gulei D, Irimie AI, Cojocneanu-Petric R, Schultze JL, Berindan-Neagoe I (2018). Exosomes-small players, big sound. Bioconjug Chem.

[17] Phinney DG, Pittenger MF (2017). Concise review: MSC-derived exosomes for cell-free therapy. Stem Cells.

[18] Chew JRJ, Chuah SJ, Teo KYW, Zhang S, Lai RC, Fu JH, Lim LP, Lim SK, Toh WS (2019). Mesenchymal stem cell exosomes enhance periodontal ligament cell functions and promote periodontal regeneration. Acta Biomater.

[19] Wu J, Chen L, Wang R, Song Z, Shen Z, Zhao Y, Huang S, Lin Z (2019). Exosomes secreted by stem cells from human exfoliated deciduous teeth promote alveolar bone defect repair through the regulation of angiogenesis and osteogenesis. ACS Biomater Sci Eng.

[20] Yáñez-Mó M, Siljander PR-M, Andreu Z, Bedina Zavec A, Borràs FE, Buzas EI, Buzas K, Casal E, Cappello F, Carvalho J (2015). Biological properties of extracellular vesicles and their physiological functions. J Extracell Ves.

[21] McGuinness D, Anthony DF, Moulisova V, MacDonald AI, MacIntyre A, Thomson J, Nag A, Davies RW, Shiels PG (2016). Microvesicles but not exosomes from pathfinder cells stimulate functional recovery of the pancreas in a mouse streptozotocin-induced diabetes model. Rejuvenation Res.

[22] Dinh PC, Paudel D, Brochu H, Popowski KD, Gracieux MC, Cores J, Huang K, Hensley MT, Harrell E (2020). Inhalation of lung spheroid cell secretome and exosomes promotes lung repair in pulmonary fibrosis. Nat Commun.

[23] Ohshima H, Wartiovaara J, Thesleff I (1999). Developmental regulation and ultrastructure of glycogen deposits during murine tooth morphogenesis. Cell Tissue Res.

[24] Yamamoto T, Domon T, Takahashi S, Anjuman KA, Fukushima C, Wakita M (2007). Mineralization process during acellular cementogenesis in rat molars: a histochemical and immunohistochemical study using fresh-frozen sections. Histochem Cell Biol.

[25] Ansari S, de Wildt BWM, Vis MAM, de Korte CE, Ito K, Hofmann S, Yuana Y (2021). Matrix vesicles: role in bone mineralization and potential use as therapeutics. Pharmaceuticals (Basel).

[26] Wuthier RE, Lipscomb GF (2011). Matrix vesicles: structure, composition, formation and function in calcification. Front Biosci (Landmark Ed).

[27] Rilla K, Mustonen AM, Arasu UT, Harkonen K, Matilainen J, Nieminen P (2019). Extracellular vesicles are integral and functional components of the extracellular matrix. Matrix Biol.

[28] Yi G, Ma Y, Chen Y, Yang X, Yang B, Tian W (2021). A Review of the functions of matrix vesicles in periodontal tissues. Stem Cells Dev.

[29] Chen NX, O'Neill KD, Moe SM (2018). Matrix vesicles induce calcification of recipient vascular smooth muscle cells through multiple signaling pathways. Kidney Int.

[30] Kunitomi Y, Hara ES, Okada M, Nagaoka N, Kuboki T, Nakano T, Kamioka H, Matsumoto T (2019). Biomimetic mineralization using matrix vesicle nanofragments. J Biomed Mater Res A.

[31] Dai XY, Zhao MM, Cai Y, Guan QC, Zhao Y, Guan Y, Kong W, Zhu WG, Xu MJ, Wang X (2013). Phosphate-induced autophagy counteracts vascular calcification by reducing matrix vesicle release. Kidney Int.

[32] Xiao Z, Camalier CE, Nagashima K, Chan KC, Lucas DA, de la Cruz MJ, Gignac M, Lockett S, Issaq HJ (2007). Analysis of the extracellular matrix vesicle proteome in mineralizing osteoblasts. J Cell Physiol.

[33] Chen NX, O'Neill KD, Chen X, Moe SM (2008). Annexin-mediated matrix vesicle calcification in vascular smooth muscle cells. J Bone Miner Res.

[34] Kapustin AN, Chatrou ML, Drozdov I, Zheng Y, Davidson SM, Soong D, Furmanik M, Sanchis P, De Rosales RT (2015). Vascular smooth muscle cell calcification is mediated by regulated exosome secretion. Circ Res.

[35] Roszkowska M, Strzelecka-Kiliszek A, Bessueille L, Buchet R, Magne D, Pikula S (2018). Collagen promotes matrix vesicle-mediated mineralization by vascular smooth muscle cells. J Inorg Biochem.

[36] Li Y, Wang J, Yue J, Wang Y, Yang C, Cui Q (2018). High magnesium prevents matrix vesicle-mediated mineralization in human bone marrow-derived mesenchymal stem cells via mitochondrial pathway and autophagy. Cell Biol Int.

[37] Mendez-Ferrer S, Scadden DT, Sanchez-Aguilera A (2015). Bone marrow stem cells: current and emerging concepts. Ann N Y Acad Sci.

[38] Yang H, Li J, Hu Y, Sun J, Guo W, Li H, Chen J, Huo F, Tian W, Li S (2019). Treated dentin matrix particles combined with dental follicle cell sheet stimulate periodontal regeneration. Dent Mater.

[39] Yang X, Ma Y, Guo W, Yang B, Tian W (2019). Stem cells from human exfoliated deciduous teeth as an alternative cell source in bio-root regeneration. Theranostics.

[40] Zhang J, Ding H, Liu X, Sheng Y, Liu X, Jiang C (2019). Dental follicle stem cells: tissue engineering and immunomodulation. Stem Cells Dev.

[41] Morsczeck C, Gotz W, Schierholz J, Zeilhofer F, Kuhn U, Mohl C, Sippel C, Hoffmann KH (2005). Isolation of precursor cells (PCs) from human dental follicle of wisdom teeth. Matrix Biol.

[42] Yang B, Chen G, Li J, Zou Q, Xie D, Chen Y, Wang H, Zheng X, Long J (2012). Tooth root regeneration using dental follicle cell sheets in combination with a dentin matrix - based scaffold. Biomaterials.

[43] Minamizaki T, Nakao Y, Irie Y, Ahmed F, Itoh S, Sarmin N, Yoshioka H, Nobukiyo A, Fujimoto C (2020). The matrix vesicle cargo miR-125b accumulates in the bone matrix, inhibiting bone resorption in mice. Commun Biol.

[44] Ansari S, BWM de Wildt, MAM Vis, CE de Korte, K Ito, S Hofmann and Y Yuana. (2021). Matrix Vesicles: Role in Bone Mineralization and Potential Use as Therapeutics. Pharmaceuticals (Basel) 14. 10.3390/ph14040289 PMC8064082 33805145

[45] Kapustin AN, Davies JD, Reynolds JL, McNair R, Jones GT, Sidibe A, Schurgers LJ, Skepper JN, Proudfoot D, Mayr M, Shanahan CM (2011). Calcium regulates key components of vascular smooth muscle cell-derived matrix vesicles to enhance mineralization. Circ Res.

[46] Balcerzak M, Malinowska A, Thouverey C, Sekrecka A, Dadlez M, Buchet R, Pikula S (2008). Proteome analysis of matrix vesicles isolated from femurs of chicken embryo. Proteomics.

[47] Shi W, Guo S, Liu L, Liu Q, Huo F, Ding Y, Tian W (2020). Small extracellular vesicles from lipopolysaccharide-preconditioned dental follicle cells promote periodontal regeneration in an inflammatory microenvironment. ACS Biomater Sci Eng.

[48] Han J, Menicanin D, Marino V, Ge S, Mrozik K, Gronthos S, Bartold PM (2014). Assessment of the regenerative potential of allogeneic periodontal ligament stem cells in a rodent periodontal defect model. J Periodontal Res.

[49] Padial-Molina M, Rodriguez JC, Volk SL, Rios HF (2015). Standardized in vivo model for studying novel regenerative approaches for multitissue bone-ligament interfaces. Nat Protoc.

[50] Liu J, Ruan J, Weir MD, Ren K, Schneider A, Wang P, Oates TW, Chang X, Xu HHK (2019). Periodontal bone-ligament-cementum regeneration via scaffolds and stem cells. Cells.

[51] Gholami L, Nooshabadi VT, Shahabi S, Jazayeri M, Tarzemany R, Afsartala Z, Khorsandi K (2021). Extracellular vesicles in bone and periodontal regeneration: current and potential therapeutic applications. Cell Biosci.

[52] Wang Y, Hu X, Zhang L, Zhu C, Wang J, Li Y, Wang Y, Wang C, Zhang Y, Yuan Q (2019). Bioinspired extracellular vesicles embedded with black phosphorus for molecular recognition-guided biomineralization. Nat Commun.

[53] Hoshi K, Ozawa H (2000). Matrix vesicle calcification in bones of adult rats. Calcif Tissue Int.

[54] Hasegawa T, Yamamoto T, Tsuchiya E, Hongo H, Tsuboi K, Kudo A, Abe M, Yoshida T, Nagai T (2017). Ultrastructural and biochemical aspects of matrix vesicle-mediated mineralization. Jpn Dent Sci Rev.

[55] Hasegawa T (2018). Ultrastructure and biological function of matrix vesicles in bone mineralization. Histochem Cell Biol.

[56] Bolean M, Borin IA, Simao AMS, Bottini M, Bagatolli LA, Hoylaerts MF, Millan JL, Ciancaglini P (2017). Topographic analysis by atomic force microscopy of proteoliposomes matrix vesicle mimetics harboring TNAP and AnxA5. Biochim Biophys Acta Biomembr.

[57] Vimalraj S (2020) Alkaline phosphatase: structure, expression and its function in bone mineralization. Gene 754:144855 10.1016/j.gene.2020.144855 32522695

[58] Bessueille L, Briolay A, Como J, Mebarek S, Mansouri C, Gleizes M, El Jamal A, Buchet R, Dumontet C, et al (2020) Tissue-nonspecific alkaline phosphatase is an anti-inflammatory nucleotidase. Bone 133:115262 10.1016/j.bone.2020.115262 PMC7185042 32028019

[59] Müller WEG, Wang S, Neufurth M, Kokkinopoulou M, Feng Q, Schröder HC, Wang X (2017). Polyphosphate as a donor of high-energy phosphate for the synthesis of ADP and ATP. J Cell Sci.

[60] Qi X, Zhang J, Yuan H, Xu Z, Li Q, Niu X, Hu B, Wang Y, Li X (2016). Exosomes secreted by human-induced pluripotent stem cell-derived mesenchymal stem cells repair critical-sized bone defects through enhanced angiogenesis and osteogenesis in osteoporotic rats. Int J Biol Sci.

[61] Seciu AM, Craciunescu O, Stanciuc AM, Zarnescu O (2019). Tailored biomaterials for therapeutic strategies applied in periodontal tissue engineering. Stem Cells Dev.

[62] Boyan BD, Sylvia VL, Dean DD, Pedrozo H, Del Toro F, Nemere I, Posner GH, Schwartz Z (1999). 1,25-(OH)2D3 modulates growth plate chondrocytes via membrane receptor-mediated protein kinase C by a mechanism that involves changes in phospholipid metabolism and the action of arachidonic acid and PGE2. Steroids.

[63] Tacey A, Qaradakhi T, Brennan-Speranza T, Hayes A, Zulli A, Levinger I (2018). Potential role for osteocalcin in the development of atherosclerosis and blood vessel disease. Nutrients.

[64] Ducy P, Desbois C, Boyce B, Pinero G, Story B, Dunstan C, Smith E, Bonadio J, Goldstein S (1996). Increased bone formation in osteocalcin-deficient mice. Nature.

[65] De Toni L, Di Nisio A, Rocca MS, De Rocco Ponce M, Ferlin A, Foresta C (2017). Osteocalcin, a bone-derived hormone with important andrological implications. Andrology.

[66] Kaddour H, Panzner TD, Welch JL, Shouman N, Mohan M, Stapleton JT, Okeoma CM (2020). Electrostatic surface properties of blood and semen extracellular vesicles: implications of sialylation and HIV-induced changes on EV internalization. Viruses.

[67] Midekessa G, Godakumara K, Ord J, Viil J, Lattekivi F, Dissanayake K, Kopanchuk S, Rinken A, Andronowska A (2020). Zeta potential of extracellular vesicles: toward understanding the attributes that determine colloidal stability. ACS Omega.

[68] Soares Martins T, Catita J, Martins Rosa I, ABdCES O, Henriques AG. Exosome isolation from distinct biofluids using precipitation and column-based approaches. PLoS One 2018;13:e0198820. 10.1371/journal.pone.0198820 PMC5995457 29889903

[69] Bhattacharjee S (2016). DLS and zeta potential: what they are and what they are not?. J Control Release.

[70] Gdyczynski CM, Manbachi A, Hashemi S, Lashkari B, Cobbold RS (2014). On estimating the directionality distribution in pedicle trabecular bone from micro-CT images. Physiol Meas.

[71] Watson PH, Watson AJ, Hodsman AB (1996). Expression of growth factor ligand and receptor genes in rat cancellous bone trabeculae and marrow. J Mol Endocrinol.

[72] Bauer W, Aub JC, Albright F (1929). Studies of calcium and phosphorus metabolism : V. A study of the bone trabeculae as a readily available reserve supply of calcium. J Exp Med.

[73] Swartz SM, Parker A, Huo C (1998). Theoretical and empirical scaling patterns and topological homology in bone trabeculae. J Exp Biol.

[74] Sela J, Schwartz Z, Amir D, Swain L, Boyan and mineral BJB. The effect of bone injury on extracellular matrix vesicle proliferation and mineral formation. 1992;17:163–7. 10.1016/0169-6009(92)90729-w 1611303

[75] Di Paolo G, De Camilli P (2006). Phosphoinositides in cell regulation and membrane dynamics. Nature.

[76] Ma JX, Wang B, Ding CF, Li HS, Jiang XJ, Wang CY, Yu J, Chen WQ. Couplet medicines of leech and centipede granules improve erectile dysfunction via inactivation of the CaSR/PLC/PKC signaling in streptozotocin-induced diabetic rats. (2020); Biosci Rep 40. 10.1042/BSR20193845 PMC7000366 31922200

[77] Mebarek S, Abousalham A, Magne D, Do le D, Bandorowicz-Pikula J, Pikula S, Buchet R. Phospholipases of mineralization competent cells and matrix vesicles: roles in physiological and pathological mineralizations. Int J Mol Sci 2013;14:5036–129. 10.3390/ijms14035036 PMC3634480 23455471

[78] Liu G, Cao W, Jia G, Zhao H, Chen X, Wang J (2018). Calcium-sensing receptor in nutrient sensing: an insight into the modulation of intestinal homoeostasis. Br J Nutr.

[79] Doroudi M, Chen J, Boyan BD, Schwartz Z (2014). New insights on membrane mediated effects of 1alpha,25-dihydroxy vitamin D3 signaling in the musculoskeletal system. Steroids.

[80] Wu LN, Genge BR, Kang MW, Arsenault AL, Wuthier RE (2002). Changes in phospholipid extractability and composition accompany mineralization of chicken growth plate cartilage matrix vesicles. J Biol Chem.

[81] Robbins PD, Morelli AE (2014). Regulation of immune responses by extracellular vesicles. Nat Rev Immunol.

[82] Shen Z, Kuang S, Zhang Y, Yang M, Qin W, Shi X, Lin Z (2020). Chitosan hydrogel incorporated with dental pulp stem cell-derived exosomes alleviates periodontitis in mice via a macrophage-dependent mechanism. Bioact Mater.

[83] Nakao Y, Fukuda T, Zhang Q, Sanui T, Shinjo T, Kou X, Chen C, Liu D, Watanabe Y (2021). Exosomes from TNF-alpha-treated human gingiva-derived MSCs enhance M2 macrophage polarization and inhibit periodontal bone loss. Acta Biomater.

[84] Li W, S Zhu, J Li, Y Huang, R Zhou, X Fan, H Yang, X Gong, NT Eissa, et al. (2011). A hepatic protein, fetuin-A, occupies a protective role in lethal systemic inflammation. PLoS One 6:e16945. 10.1371/journal.pone.0016945 PMC3035675 21347455

[85] Bozycki L, Mroczek J, Bessueille L, Mebarek S, Buchet R, Pikula S, Strzelecka-Kiliszek A. Annexins A2, A6 and Fetuin-A affect the process of mineralization in vesicles derived from human osteoblastic hFOB 1.19 and osteosarcoma saos-2 cells. 2021;Int J Mol Sci 22:3993. 10.3390/ijms22083993 PMC8069967 33924370

