# Supplementary material for: Matrix vesicles from dental follicle cells improve alveolar bone regeneration via activation of the PLC/PKC/MAPK pathway
Source: Stem Cell Res Ther. 2022 Jan 29;13:41. doi: 10.1186/s13287-022-02721-6 (PMC8800263; doi:10.1186/s13287-022-02721-6)
Supplement: Supplementary file 1 — Additional file 1: Figure S1. Characterization of dental follicle cells (DFCs). Figure S2. The creation of the alveolar bone defects in the SD rat guided by a module. [file 13287_2022_2721_MOESM1_ESM.docx]

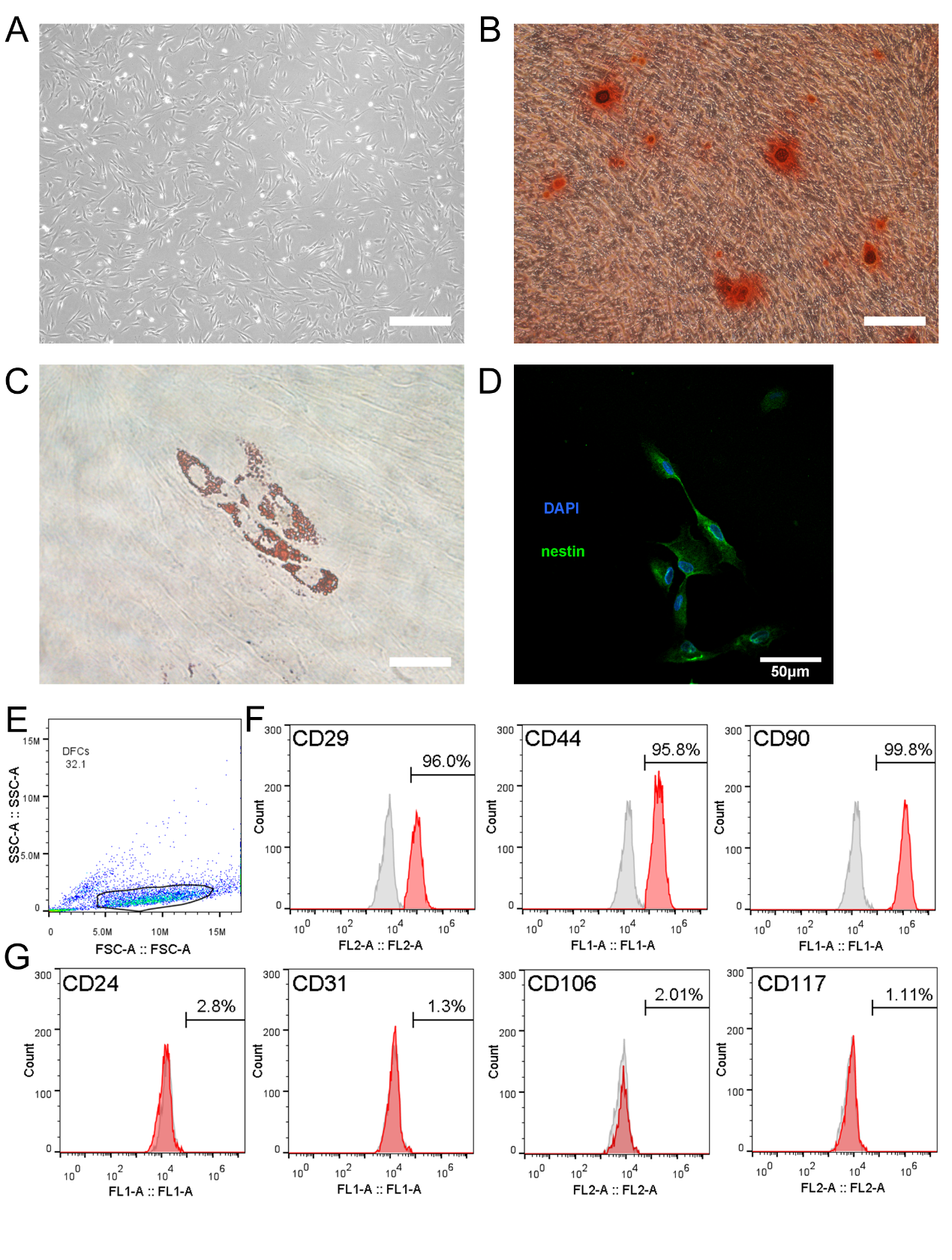


Supplementary Figure 1. Characterization of dental follicle cells (DFCs). (A) The typical morphology of mesenchymal cells with spindle shapes was observed in DFCs. Scale bars: 500 μm. (B-D) Detection of Multi-differentiation of DFCs. (B) Osteogenic differentiation. Representative images of alizarin red s staining of DFCs with mineral nodules after osteogenic culturing for 14 days. Scale bars: 500 μm. (C) Adipogenic differentiation. Representative images of oil red o staining of DFCs with lipid clusters after adipogenic induction for 24 days. Scale bars: 50 μm. (D) Neurogenic differentiation. Representative images of immunofluorescence staining of DFCs with neurogenic differentiation marker (nestin; green) and nuclei (DAPI; blue). Scale bars: 50 μm. (E-G) Flow-cytometry analysis of the surface markers of DFCs. Red waves represent the experimental groups, and grey waves indicate the control. (E) The strategy of gating for flow-cytometry analysis. (F) Positive markers involve CD29, CD44, CD90. (G) Negative markers including CD24, CD31, CD106, and CD117.


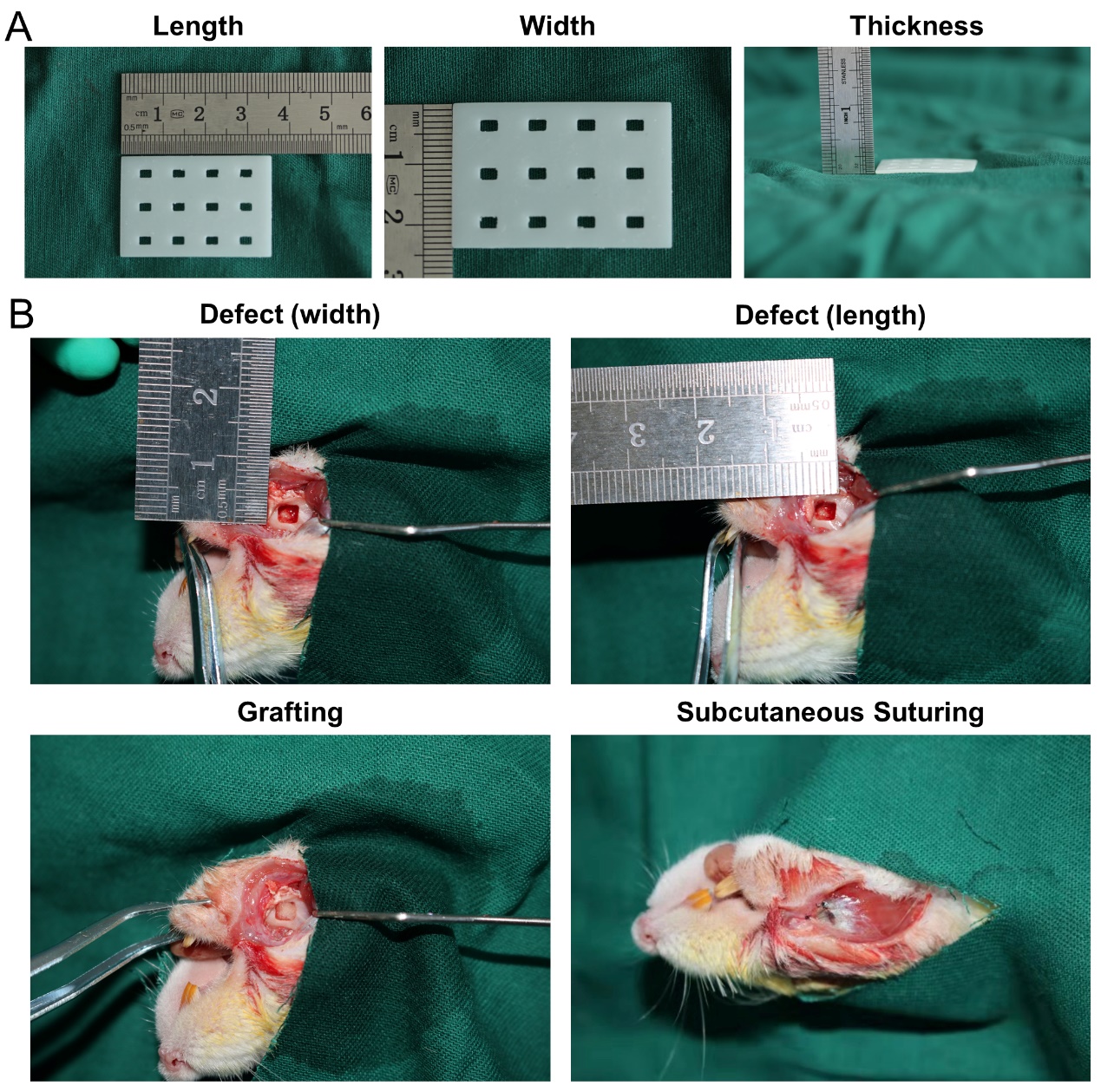


Supplementary Figure 2. The creation of the alveolar bone defects in the SD rat guided by a module. (A) The integral module was designed with 12 holes and measured at 32 mm (Length) ×24 mm (Width) ×1 mm (Thickness). (B) Records during operation showed the created defects were about 3 mm (Length) ×2 mm (Width) ×1 mm (Depth) in size.
